# Resistance of first-line targeted drugs in hepatocellular carcinoma: the epigenetic regulation mechanisms

**DOI:** 10.1038/s41419-025-08105-x

**Published:** 2025-11-26

**Authors:** Ding Qi, Yongqing Qin, Haidong Zhu, Yong Li, Shisong Han

**Affiliations:** 1 Guangdong Provincial Key Laboratory of Tumor Interventional Diagnosis and Treatment, Zhuhai Institute of Translational Medicine, Zhuhai People’s Hospital (Zhuhai Clinical Medical College of Jinan University), Beijing Institute of Technology, Zhuhai, Guangdong China; 2 Center of Interventional Radiology and Vascular Surgery, Department of Radiology, Basic Medicine Research and Innovation Center of Ministry of Education, Zhongda Hospital, Medical School, Southeast University, Nanjing, China

**Keywords:** Cancer epigenetics, Pharmacology

## Abstract

Targeted therapy has revolutionized the treatment landscape of hepatocellular carcinoma (HCC), offering unprecedented hope to patients. However, despite its promise, significant challenges arise in the form of drug resistance. Only a fraction of HCC patients respond to targeted therapy, and even those who respond often develop resistance over time. Sorafenib and lenvatinib, the sole first-line targeted therapeutic drugs for HCC, face severe clinical limitations due to drug resistance. Understanding the mechanisms underlying sorafenib/lenvatinib resistance is crucial for maximizing treatment efficacy. Recent studies have highlighted the pivotal role of epigenetic regulation in drug resistance. Cancer is recognized as both a genetic and epigenetic disease, with epigenetic factors influencing various aspects of tumor cell biology, especially drug resistance. This review systematically summarizes the mechanisms of epigenetic-mediated sorafenib/lenvatinib resistance, encompassing non-coding RNA (ncRNA) regulation, DNA methylation, RNA methylation, and histone modification. These abnormal epigenetic processes typically influence biological activities, including escaped programmed cell death, tumor metabolic reprogramming, formation and maintenance of drug-resistant cells, uncontrolled cell proliferation signaling pathways, and abnormal transport processes, ultimately culminating in profound drug resistance. By comprehensively summarizing the latest discoveries in epigenetic regulation mechanisms, this review highlights potential strategies to overcome drug resistance, paving the way for future advancements in HCC treatment.

## Facts

- Epigenetic regulation plays a significant role in sorafenib/lenvatinib resistance.
- Epigenetic drug resistance outcomes involve programmed cell death evasion, metabolic reprogramming, the formation and maintenance of drug-resistant cells, uncontrolled cell proliferation signaling pathways, and abnormal transport processes.
- Combining epigenetic therapy with targeted drugs demonstrates potential in reversing drug resistance.
- Personalized therapy represents a promising strategy to enhance the effectiveness of clinical drug resistance treatments.

## Open questions

- Whether the currently reported drug resistance mechanisms show significant differences in clinical patients?
- Can resistance-overcoming therapies validated in animal studies achieve comparable efficacy in clinical patients?
- Could targeting the shared resistance mechanisms/targets of sorafenib and lenvatinib simultaneously reverse resistance to both drugs?
- How can we address the challenges in overcoming resistance posed by the complexity of epigenetic drug resistance?

## Introduction

Primary liver cancer is the third most common cause of cancer-related death worldwide, with hepatocellular carcinoma (HCC) accounting for over 80% of all cases [1]. In stark contrast to other prevalent cancers, such as breast, lung, and prostate, which are experiencing declining mortality rates, HCC-related mortality continues to climb at a rate of 2–3% annually [2]. Projections indicate that the global tally of liver cancer instances will surpass 1 million by 2025, with the highest incidence rates reported in East and Southeast Asia, as well as Central and West Africa [2, 3].

To address the substantial impact of HCC, modern medicine offers various therapeutic options. Surgical resection and liver transplantation have the potential to completely cure early-stage HCC with 5-year survival rates exceeding 60% for both treatments [4]. For HCC patients who do not qualify for surgery, interventional techniques such as ablative therapy [5] and transcatheter arterial chemoembolization (TACE) [6] provide some survival benefit. However, the eligibility criteria for these treatments often clash with the characteristics of advanced HCC. When the aforementioned treatments are unavailable, systemic therapy becomes the primary choice [7], encompassing chemotherapy, targeted therapy, and immunotherapy [8].

In the past decade, there has been significant progress in targeted therapy for the treatment of mid-stage and advanced HCC, prolonging patient survival. Targeted therapy drugs mainly include multikinase inhibitors and vascular endothelial growth factor (VEGF) receptor antibodies. Sorafenib and lenvatinib are currently the only first-line targeted drugs for HCC treatment [9]. Importantly, sorafenib is efficacious in ~30% of patients [10]; while lenvatinib demonstrates a similarly modest overall response rate of 24% [11]. High resistance rates to both sorafenib and lenvatinib pose significant impediments to achieving therapeutic success in the treatment of HCC. Available evidence suggests that numerous resistance cases arise due to aberrant epigenetic regulation. Consequently, it is imperative to conduct thorough analyses of the underlying mechanisms responsible for this resistance and to identify effective strategies aimed at delaying or overcoming drug resistance, ultimately improving the prognosis for HCC patients.

Previous reviews have analyzed epigenetic mechanisms in HCC drug resistance. Oura et al. classified systemic therapy resistance into epigenetic regulation and tumor microenvironment (TME) modulation, mapping drug-specific resistance networks involving molecular targeted agents (MTAs) and immune checkpoint inhibitors (ICIs) [12]. Sun et al. systematically categorized epigenetic resistance mechanisms by regulatory modalities (DNA methylation, RNA regulation, histone modification, chromatin remodeling), emphasizing epigenetic-targeted agents (e.g., DNMT/HDAC inhibitors) and their clinical potentia [13]. While Oura et al. emphasize epigenetic-TME interplay in resistance networks, Sun et al. focus on mechanistic insights and clinical translation of epigenetic therapies, offering complementary frameworks to address HCC resistance. Our work attempts to both extend the reported epigenetic-mediated resistance mechanism and delve deeper into the clinical translational potential of epigenetic therapies in overcoming resistance, thereby providing novel insights into therapeutic development.

**Fig. 1:**
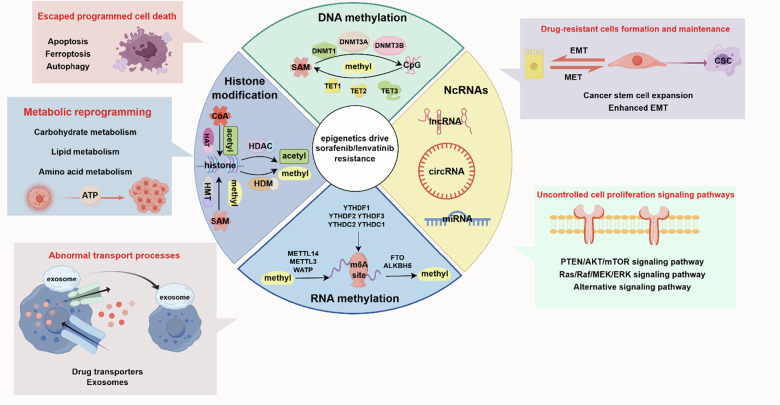
Schematic diagram of epigenetic regulation mechanisms and drug resistance outcomes. The mechanisms of epigenetic regulation contributing to drug resistance include: DNA/RNA methylation, histone modifications, and non-coding RNA regulation; while the consequential manifestations of drug resistance encompass: escaped programmed cell death, tumor metabolic reprogramming, formation and maintenance of drug-resistant cells, uncontrolled cell proliferation signaling pathways and abnormal transport processes. Image created by Figdraw.

In this comprehensive review, we will delve into the epigenetic regulatory mechanisms that confer resistance to sorafenib and lenvatinib, and provide an in-depth analysis of the potential of epigenetic therapy and its clinical translation. Specifically, we will categorize the mechanisms underlying epigenetic modulation-induced drug resistance based on four primary epigenetic regulatory means: ncRNA regulation, DNA methylation, RNA methylation, and histone modification. These mechanisms will be grouped into the following categories: escaped programmed cell death, tumor metabolic reprogramming, formation and maintenance of drug-resistant cells, uncontrolled cell proliferation signaling pathways, and abnormal transport processes (Fig. 1).

## Epigenetic modification

The term “epigenetics” first introduced by Conrad Waddington, refers to stable genetic phenotypes that arise from chromosomal alterations without altering the fundamental DNA sequence [14]. Epigenetic mechanisms determine the transcriptional availability of various parts of the genome, thereby shaping long-term cell behavior [15]. The most extensively studied epigenetic regulations encompass direct DNA methylation, post-translational modifications of histones, and regulations mediated by ncRNA, collectively forming an intricate regulatory network; aberrant regulatory patterns of tumor cells within this network can directly lead to drug resistance [16]. In the past decade, dysregulated RNA methylation has also been identified in human cancers [17]. This dysregulation promotes tumor resistance by modifying the stability of crucial gene transcripts and influencing certain signaling pathways [18]. Below, we will briefly introduce the key modes of epigenetic regulation, DNA methylation, histone modification, ncRNA regulation, and RNA methylation. These regulatory mechanisms collectively contribute to the development of drug resistance against molecularly targeted therapies through intricate biological processes.

### NcRNAs

Over the past decades, ncRNAs have transitioned from being considered “junk products” to essential regulatory molecules that modulate gene expression. Despite not encoding proteins, ncRNAs exert influence on gene expression patterns through diverse mechanisms, encompassing chromatin remodeling, transcription, post-transcriptional modification, and signal transduction [19]. The three main types of ncRNAs are microRNAs (miRNAs), long non-coding RNAs (lncRNAs), and circular RNAs (circRNAs), each playing a distinct role in cancer. MiRNAs are short RNAs (~22 nucleotides [nt]); MiRNAs modulate gene expression through their association with the RNA-induced silencing complex (RISC), a multiprotein entity. This complex subsequently binds to the 3’ untranslated region of the target mRNA, leading to the inhibition of translation, the shortening of the mRNA’s poly (A) tail, and its eventual degradation [20]. LncRNAs and circRNAs are longer (>200 nt); lncRNAs are linear, whereas circRNAs are cyclic [21]. The dysregulation of lncRNA is involved in tumor progression by regulating chromatin regulation and transcription, sponging miRNAs, and affecting structural functions [22]. Some circRNAs function similarly to lncRNAs, but many of their mechanisms remain unclear [23]. They are generated through back-splicing of pre-mRNA, forming a covalently closed loop without a 5’-cap or a 3’-poly (A) tail. Compared to linear RNA, circRNA is less susceptible to degradation by exogenous enzymes and exhibits higher stability. The physiological effects of circRNA are often mediated through binding to specific miRNAs (sponging), thereby eliminating the inhibitory effect of the miRNA on mRNA translation [24]. These ncRNAs classes are closely linked to various cancer processes, including therapeutic resistance.

### DNA methylation

The core of DNA methylation involves transferring a methyl group from S-adenosylmethionine (SAM) to the C5 position of cytosine in cytosine-phosphate-guanine (CpG) dinucleotides, a process facilitated by DNA methyltransferase (DNMT) enzymes, resulting in the formation of 5-methylcytosine; CpG dinucleotides are unevenly distributed in mankind genomes, predominantly clustering in CpG islands (CGIs), which are short, interspersed DNA sequences that deviate significantly from the typical genomic pattern and are characterized by their abundance of unmethylated CpG dinucleotides. It is plausible that the majority, or even all, of these CGIs function as sites for transcription initiation. In normal somatic promoters, methylation of CGIs can result in gene silencing, either directly by impeding the binding of transcription factors or indirectly through the interaction of 5-methylcytosine (5mC) with methyl-CpG binding domain (MBD) proteins [25]. However, in tumor cells, abnormalities frequently arise in the DNA methylation process, contributing to tumorigenesis and progression. Disruptions to these processes or abnormalities in the participating enzymes can silence tumor suppressor genes [26, 27]. Key enzymes involved in these processes include DNA methyltransferases (e.g., DNMT1, DNMT3A, and DNMT3B) and DNA demethylases such as ten-eleven translocation 1 (TET1), TET2, and TET3 [28]. Methyltransferase abnormalities usually result in hypermethylation of tumor suppressor gene promoters, silencing those genes. Conversely, demethylase abnormalities cause hypomethylation of oncogene promoters, leading to drug resistance [29].

### Histone modification

Histone is the core component of the nucleosome subunit, featuring a side chain or tail with a dense distribution of basic lysine and arginine residues. The histone tail undergoes extensive cooperative covalent post-translational modifications (PTMs) to regulate chromatin states. The best-characterized and most common types are acetylation and methylation. Chromatin-modifying enzymes are classified as histone acetyltransferases (HATs), histone deacetylases (HDACs), histone methyltransferases (HMTs), and histone demethylases (HDMs) [30]. Generally, histone acetylation catalyzed by histone acetyltransferases (HATs) is associated with transcriptional activation, while hypoacetylation by histone deacetylases is linked to transcriptional repression. Histone methylation is relevant to both active and silent genes. For instance, trimethylation of lysine 27 on histone H3 (H3K27) serves as a silencing mark, while methylation of lysine 4 on histone H3 (H3 K4) is found at the promoters of active genes. Histone demethylation, on the other hand, is usually associated with silent genes [20, 30]. These modifications control gene expression via dynamic addition and removal, influencing processes such as transcriptional activation, chromosome folding, and DNA repair [31].

### RNA methylation

Emerging evidence suggests that dysregulated RNA methylation is associated with cancer progression in humans [32]. Thus far, more than 100 types of RNA modifications have been identified [33]. The most prevalent type of methylation, N6-methyladenosine (m6A), occurs at the N6 position of adenosine and represents over 80% of RNA methylation in eukaryotes. Three types of proteins control m6A modification. The first type comprises m6A methyltransferases, including methyltransferase-like 3 (METTL3), METTL14, and Wilms tumor 1-associated protein (WTAP). METTL14 forms a stable complex with METTL3; WTAP ensures that the METTL3-METTL14 heterodimer is located in the nucleus and maintains catalytic activity. The second type comprises m6A demethylases, such as AlkB homolog H5 (ALKBH5) and fat mass and obesity-associated protein (FTO) binding proteins, which are mainly responsible for the reversal of existing m6A modifications. The third type comprises methylation recognition proteins; notable examples are the YT521-B homology (YTH) domain proteins (YTH domain-containing family proteins 1 (YTHDF1), YTHDF2, YTHDF3, YTHDC1, YTHDC2). These proteins primarily recognize and bind m6A sites on RNA, thereby altering processes such as splicing, localization, translation, and stability [34, 35].

## Epigenetic modifications involved in sorafenib resistance

Sorafenib, an oral multikinase inhibitor targeting VEGF receptors, RAF, and platelet-derived growth factor (PDGF) receptors, exerts anti-angiogenic and antitumor effects [36]. The SHARPE trial revealed that patients treated with oral sorafenib exhibited significantly better overall survival (OS) compared to those receiving a placebo, with OS extending to 10.7 months versus 7.9 months (hazard ratio [HR]: 0.69; confidence interval [CI]: 0.55–0.87; *P* < 0.001). In addition, the time to radiographic progression was prolonged by nearly 3 months with sorafenib (5.5 months vs. 2.8 months; HR: 0.58; CI: 0.45–0.74; *P* < 0.001) [37]. A phase III randomized, double-blind, placebo-controlled trial conducted in the Asia-Pacific region further confirmed that sorafenib significantly improved survival in patients with advanced HCC [38]. Consequently, in 2008, sorafenib was approved by the FDA as a first-line targeted therapy for HCC [39]. This review specifically focuses on the role of epigenetic modifications in sorafenib resistance.

### Epigenetic modification enables escaped programmed cell death

Tumor cell death pathways include unprogrammed cell death and programmed cell death (PCD). Unprogrammed cell death is a passive process triggered by external stimuli and lacks cellular regulation. Conversely, PCD is a form of regulated cell death controlled by a complex gene network [40]. Recent advancements have significantly enhanced our understanding of how epigenetic modifications contribute to sorafenib resistance through aberrant PCD. Most tumor cells can partially evade PCD through epigenetic modifications. PCD in tumor cells involves several crucial escape mechanisms associated with sorafenib resistance, including apoptosis, ferroptosis, and autophagy pathways [41].

#### Apoptosis

**Fig. 2:**
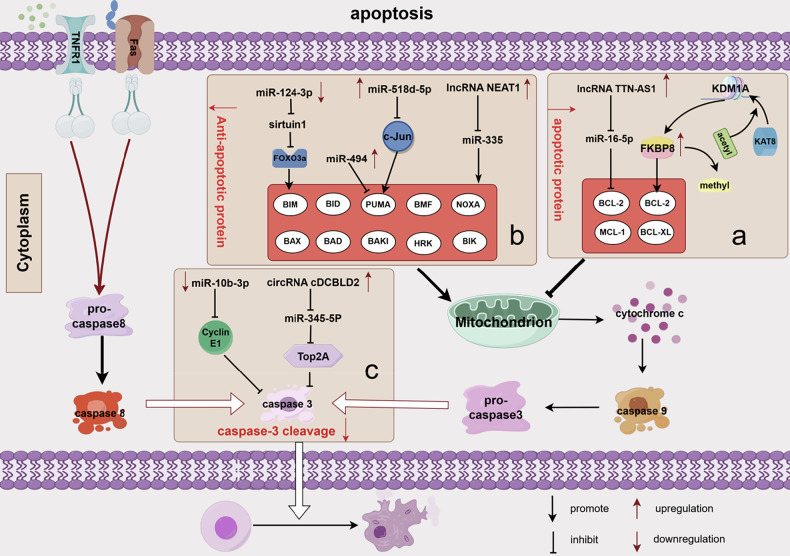
Epigenetic modifications enhance sorafenib resistance by inhibiting apoptosis, involving the following regulators. **a** KDM1A and lncRNA TTN-AS1 promote sorafenib resistance by upregulating anti-apoptotic proteins. **b** miR-494, lncRNA NEAT1, miR-124-3p, and miR-518d-5p promote resistance by suppressing pro-apoptotic proteins. **c** miR-10b-3p, circRNAs, and cDCBLD2 promote resistance by directly inhibiting caspase-3 cleavage. Image created by Figdraw.

Apoptosis, the predominant form of cell death, is a critical mechanism triggered by chemotherapy and molecular targeting therapies [42]. Apoptosis comprises endogenous and exogenous pathways. The endogenous apoptotic pathway results from increased mitochondrial permeability and the cytoplasmic release of pro-apoptotic molecules, such as cytochrome c. Caspase-9 responds to cytochrome c accumulation in the cytoplasm by forming apoptosomes and initiating caspase-mediated PCD. This process is tightly regulated by the B-cell lymphoma-2 (BCL-2) protein family, which includes pro-apoptotic members (BAX, BAK1, BID, BIM, PUMA, BAD, BIK, BMF, NOXA, and HRK) and anti-apoptotic members (BCL-2, BCL-XL, BCL-W, and MCL1). Anti-apoptotic proteins inhibit the release of cytochrome c from mitochondria, whereas pro-apoptotic proteins function by promoting this release [43, 44]. Caspase-3, a downstream effector shared by both pathways, cleaves deoxyribonucleic acid to induce apoptosis [45]. Since sorafenib primarily acts through the endogenous apoptosis pathway, we focus on how abnormalities in this pathway mediate sorafenib resistance (Fig. 2).

##### Anti-apoptotic protein-induced resistance

Increased levels of anti-apoptotic proteins are partly attributed to epigenetic regulation of the KDM1A (lysine demethylase 1A)/FKBP8 (FKBP prolyl isomerase 8)/BCL-2 (B-cell lymphoma-2) axis. The cytoplasmic localization and protein stability of KDM1A were promoted by acetylation at lysine-117 by the acetyltransferase KAT8. The stably expressed KDM1A demethylates FKBP8 in the cytoplasm, upregulating expression of the anti-apoptotic protein BCL-2 [46]. Zhou et al. also described a mechanism where the lncRNA TTN-AS1 inhibits miR-16-5p, thereby activating the phosphatase and tensin homolog (PTEN)/protein kinase B (Akt) signaling pathway and upregulating BCL-2 protein expression. Overexpression of BCL-2 protein enhances HCC resistance to sorafenib [47].

##### Decreased apoptotic protein-induced resistance

MiR-494, overexpressed in HCC, induces sorafenib resistance by suppressing the levels of apoptosis modulator proteins. Specifically, the reduction in p53 upregulated modulator of apoptosis (PUMA) protein expression results in decreased caspase activity, effectively inhibiting apoptosis [48]. Elevated circulating levels of miR-518d-5p have been associated with shorter sorafenib treatment duration and reduced OS. MiR-518d-5p mediated mitochondrial dysfunction, leading to the silencing of c-Jun and the obstruction of PUMA transcription. Consequently, the downregulation of PUMA significantly diminishes HCC sensitivity to sorafenib and inhibits the production of reactive oxygen species (ROS), further decreasing apoptosis in cancer cells [49]. The lncRNA NEAT1 is also highly expressed in HCC cells. High of NEAT1 levels can downregulate miR-335 expression thereby activating the downstream c-Met-Akt pathway and reducing the production of apoptotic proteins. This mechanism counteracts sorafenib-induced apoptosis and contributes to drug resistance [50]. Dong et al. observed that miR-124-3p expression is downregulated in HCC tissues; this downregulation enhanced the dephosphorylation of AKT2 and silent information regulator sirtuin 1 (SIRT1 or sirtuin 1), resulting in decreased downstream forkhead box class O3a (FOXO3a) activity. Although FOXO3a generally plays a pro-apoptotic role via BIM promoter activation, its suppression reduces cellular sensitivity to sorafenib-induced apoptosis [51].

##### Inhibited caspase-3 cleavage-induced resistance

Shao et al. conducted a comparative analysis of differential miRNA expression between sorafenib-sensitive and sorafenib-resistant HCC cells, revealing a significant downregulation of miR-10b-3p in the resistant cell lines. This depletion of miR-10b-3p inhibits caspase-3 cleavage by upregulating cyclin E1, thereby reducing the capacity of sorafenib to induce apoptosis in HCC cells and ultimately leading to drug resistance [52]. The circRNA cDCBLD2, highly expressed in HCC, promotes HCC cell survival during sorafenib treatment. By competitively binding to miR-345-5p, cDCBLD2 enhances the stability of DNA topoisomerase IIα (TOP2A) mRNA. Normally, miR-345-5p directly targets the TOP2A coding sequence to promote caspase-3 activation and induce apoptosis. However, in the presence of overexpressed cDCBLD2, this process is reversed, allowing the cells to acquire resistance to sorafenib [53].

#### Ferroptosis

**Fig. 3:**
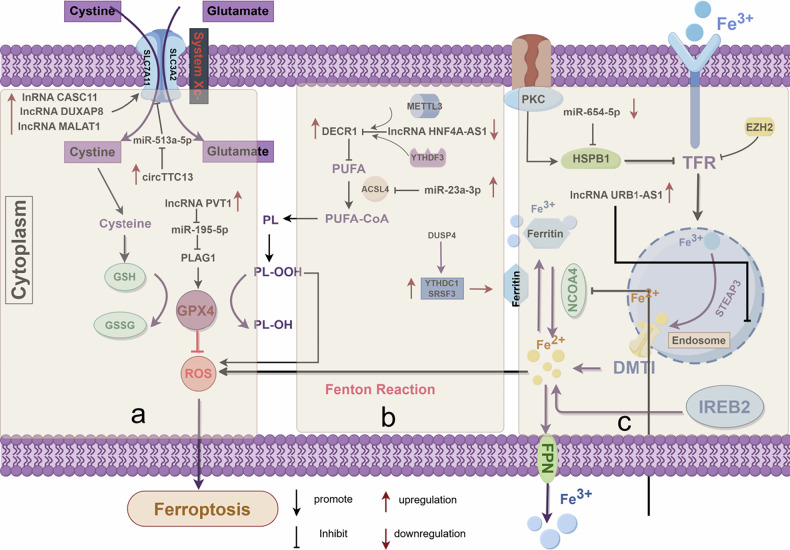
Epigenetic modifications contribute to sorafenib resistance by suppressing ferroptosis, involving the following regulators. **a** lncRNA CASC11, lncRNA DUXAP860, lncRNA MALAT1, circTTC13, and lncRNA PVT1 inhibit ferroptosis by targeting the System Xc−/GPX4 pathway. **b** miR-23a-3p and lncRNA HNF4A-AS1 suppress ferroptosis by targeting the lipid peroxidation pathway. **c** miR-654-5p, lncRNA URB1-AS1, EZH2, and YTHDC1 suppress ferroptosis by targeting the iron metabolism pathway with reduced intracellular free Fe²⁺ levels. Image created by Figdraw.

Ferroptosis represents a newly identified form of PCD that is induced by iron-dependent oxidative stress. Whereas apoptosis is triggered by caspase activity, ferroptosis is induced by ROS, which are byproducts of mitochondrial oxidative phosphorylation [54]. ROS include superoxide anion (O_2_^•-^), hydroxyl radical (•OH), hydrogen peroxide (H_2_O_2_), and singlet oxygen (^1^O_2_) [55]. Despite the higher metabolic rate of cancer cells leading to a greater ROS load and making them more susceptible to ferroptosis compared to normal cells, cancer cells employ epigenetic regulation to counteract the increases in ferroptosis caused by their high metabolism. This enhances their survival under oxidative stress and promotes resistance to sorafenib [56]. Ferroptosis centers around three pathways: system xc−/glutathione peroxidase 4 (GPX4), lipid peroxidation, and iron metabolism. Epigenetic regulatory factors promote sorafenib resistance by modifying these processes to inhibit ferroptosis [57] (Fig. 3).

##### System Xc−/GPX4 pathway

System Xc− is a heterodimeric transporter composed of two subunits, solute carrier family 7 member 11 (SLC7A11) and solute carrier family 3 member 2 (SLC3A2). Its primary function is to mediate the exchange of extracellular cystine for intracellular glutamate. Upon uptake, cystine is reduced to cysteine, which serves as a key precursor for glutathione (GSH) synthesis. GPX4 inhibits ROS production by converting glutathione (GSH) to oxidized glutathione (GSSG) and reducing the cytotoxic lipid peroxide (L–OOH) to the corresponding alcohol (L–OH) [58]. Inhibition of System Xc- impairs cystine uptake, leading to GSH depletion, decreased GPX4 activity, and accumulation of lipid peroxides—ultimately triggering ferroptosis.

Sorafenib induces ferroptosis by inhibiting system xc− activity [59]. Consequently, some epigenetic regulators promote sorafenib resistance within HCC by restoring system xc− activity. In HCC, the overexpression of lncRNA CASC11 [60] and lncRNA DUXAP8 [61] suppresses ferroptosis by upregulating SLC7A11 levels. Shi et al. uncovered an NSUN2/MALREF-mediated m5C methylation axis promoting lncRNA metastasis-associated lung adenocarcinoma transcript 1 (MALAT1) expression in HCC. MALAT1 suppresses ferroptosis by binding ELAVL1 to enhance its cytoplasmic translocation, thereby stabilizing SLC7A11. Importantly, the combination of MALAT1-IN1(MALAT1 inhibitor) with sorafenib demonstrated enhanced antitumor efficacy in preclinical HCC models [62]. Furthermore, circTTC13 functions as a sponge for miR-513a-5p, indirectly elevating SLC7A11 expression to inhibit ferroptosis [24]. Cytoplasmic lncRNA PVT1 competitively binds miR-195-5p to upregulate Pleomorphic Adenoma Gene 1 (PLAG1), subsequently increasing GPX4 expression. Pharmacological PLAG1 inhibition demonstrated synergistic effects with sorafenib across preclinical HCC models, though functional characterization of the PVT1/miR-195-5p/PLAG1 axis in ferroptosis modulation remains incomplete due to observed phenotypic heterogeneity [63].

##### The lipid peroxidation pathway

In ferroptosis, polyunsaturated fatty acid (PUFA) is activated into PUFA-CoA by Acyl-CoA synthetase long-chain family 4 (ACSL4) and subsequently esterified into phospholipid (PL)-PUFA complexes via LPCAT3. Under iron-dependent catalysis by lipoxygenases (LOXs) or ROS, PL-PUFA undergoes peroxidation to generate phospholipid hydroperoxides (PL–OOH). In the absence of GPX4-mediated detoxification, PL–OOH undergoes pathological accumulation [64].

ACSL4 is an important regulator of fatty acid metabolism and a key determinant of cellular sensitivity to ferroptosis [65]. ACSL4 and GPX4, respectively, regulate ROS accumulation in positive and negative directions [66]. ACSL4 inhibition leads to drug resistance in HCC. For example, Lu et al. observed a greater than tenfold increase in miR-23a-3p expression within sorafenib-resistant cells relative to the parental cell line. The upregulation of miR-23a-3p directly inhibits ferroptosis by suppressing ACSL4, blocking sorafenib sensitivity in HCC [67]. In addition, depletion of PUFA contributes to sorafenib resistance. Zhao et al. demonstrated a time-dependent decrease in lncRNA HNF4A-AS1 expression during prolonged sorafenib treatment, potentially mediated by HIF-1α activation under hypoxic stress. HNF4A-AS1 destabilizes DECR1 mRNA via METTL3-mediated m6A methylation with YTHDF3 as the reader protein. However, HNF4A-AS1 deficiency leads to DECR1 overexpression, accelerating PUFA oxidation to deplete PUFA reservoirs and confer sorafenib resistance [68].

##### The iron metabolism pathway

Iron metabolism during ferroptosis comprises the following pathway: extracellular Fe^3+^ is transported into cells via the transferrin–transferrin receptor complex [69], then reduced to Fe^2+^ by the six-transmembrane epithelial antigen of prostate family member 3 (STEAP3) metal reductase in endosomes, and finally released into the cytosol through divalent metal transporter 1 (DMT1) [70]. Excess Fe^2+^ is stored as ferritin (FTH1: Oxidizes Fe²⁺ to Fe³⁺, promoting iron storage; FTL: Participates in the structural stability of ferritin and iron storage) or exported via membrane ferroportin 1 (FPN1) [71]. A fraction of intracellular Fe^2+^ participates in the formation of the unstable labile iron pool (LIP), where highly oxidized Fe2+ generates highly toxic hydroxyl-free radicals (ROS) through the Fenton reaction, effectively inducing cancer cell death [72]. HCC cells develop drug resistance by inhibiting various steps of the process through intrinsic genetic alterations.

Sun et al. discovered that downregulation of miR-654-5p led to increased levels of the downstream protein heat shock protein family B member 1 (HSPB1), which inhibits iron uptake by suppressing transferrin receptor protein 1 (TFR1) [73]. EZH2 enhances H3K27me3 in hepatocellular HCC, leading to reduced RNA polymerase II binding at the TFR2 promoter region, which ultimately results in downregulation of TFR2 expression [74]. A significant decrease in the intracellular iron content reduces free radical production from the Fenton reaction, thereby inhibiting sorafenib-induced ferroptosis and enhancing drug resistance. In addition, Gao et al. revealed that nuclear receptor coactivator 4 (NCOA4) binds ferritin for lysosomal degradation, elevating intracellular free iron. Elevated lncRNA URB1-AS1 suppresses NCOA4, thereby reducing free iron and ROS levels to inhibit sorafenib-induced ferroptosis [59]. Hao et al. identified sorafenib-induced upregulation of DUSP4 as an adaptive feedback response to MAPK pathway inhibition. Mechanistically, DUSP4-mediated phosphorylation of YTHDC1 at threonine residue T148 enhances its binding to SRSF3, thereby reducing nuclear retention of FTH1/FTL mRNA. This elevates FTH1/FTL translation efficiency, ultimately suppressing ferroptosis [75].

#### Autophagy

**Fig. 4:**
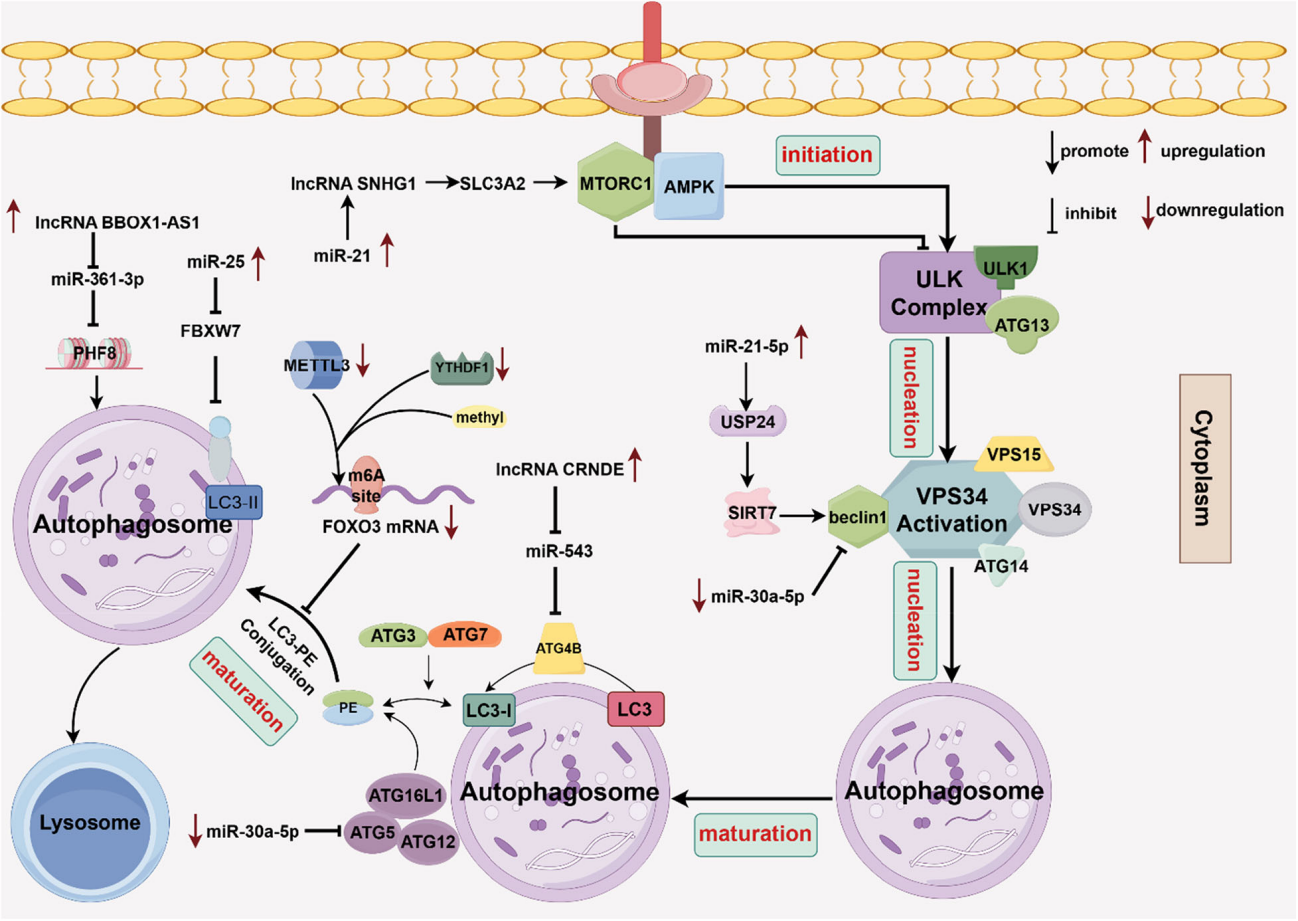
Epigenetic modification promotes sorafenib resistance by regulating autophagy. Some types of autophagy have pro-death effects and cause resistance when inhibited, whereas other types of autophagy have protective effects and cause resistance when enhanced. Classical autophagy comprises four key steps: initiation, nucleation, maturation, and degradation; epigenetic modification regulates autophagosome formation by affecting one or more of these processes. Epigenetic regulatory factors involved in the above processes include ncRNAs (e.g., miR-21, miR-25, miR-21-5p, hsa-miR30a-5p, lncRNA CRNDE, lncRNA BBOX1-AS1), RNA methylation modification enzymes (e.g., METTL3, YTHDF1), and histone demethylase (PHF8). Image created by Figdraw.

Autophagy, a self-degrading system usually accompanied by cell death (not a death mechanism), constitutes a lysosome-mediated physiological process for degrading and recycling damaged proteins and organelles. Autophagy is essential for maintaining cell homeostasis in unfavorable environments; dysregulated autophagy often poses substantial health risks with dichotomous effects (e.g., inhibiting tumor initiation while supporting tumor progression) [76, 77]. Classical autophagy encompasses a multiphasic sequence comprising four pivotal stages: initiation, nucleation, maturation, and degradation. These stages involve the sequential activation and selective recruitment of autophagy-related proteins (ATGs). Upstream regulators of autophagy include mechanistic target of rapamycin kinase 1 (mTORC1; inhibition) and adenosine monophosphate-activated protein kinase (AMPK; activation). Initiation involves activation of the ULK complex, which includes UNC-51-like kinase 1 (ULK1), ULK2, FIP200, ATG13, and ATG101. Nucleation comprises the response of autophagy-specific VPS34 complex I (including VPS34, beclin-1, ATG14, and VPS15) to the ULK complex, thereby catalyzing phosphatidylinositol-3-phosphate (PI3P) production on the autophagosome membrane. During autophagosome maturation, PI3P recruits the autophagy coupling machinery, in which the ATG5/ATG12/ATG16L1 complex guides ATG3 and ATG7 to conjugate LC3 to phosphatidylethanolamine (PE) on the membrane, forming LC3-II. ATG4 cleaves LC3 into its soluble form (LC3-Ⅰ), enabling the link between LC3 and PE. Cancer cells can epigenetically modify any of these steps to achieve drug resistance [76–78] (Fig. 4).

Li et al. found that sorafenib treatment led to miR-21 enrichment in the affected nucleus and promoted expression of the lncRNA SNHG1. SNHG1 activates the Akt/mTOR signaling pathway by promoting SLC3A2 transcription, thus inhibiting autophagy. This process decreases the capacity for autophagy to promote cell death, leading to sorafenib resistance [79, 80]. In addition, miR-25 was highly expressed in HCC tissues, where it negatively regulated the expression of the downstream suppressor gene F-box and WD repeat domain-containing 7 (FBXW7), a component of the SKP1-cullin-1-F-box-protein (SCF) E3 ligase complex involved in autophagy-related ubiquitination. MiR-25 promotes autophagy and mediates sorafenib resistance by degrading FBXW7 mRNA and upregulating LC3-II protein levels [81]. Hu et al. found that compared with neighboring tissues, HCC tissues showed increased miR-21-5p expression. miR-21-5p promoted the expression of ubiquitination-specific peptidase 24 (USP24); the interaction between USP24 and SIRT7 decreased the level of SIRT7 ubiquitination in HCC cells, while upregulating the LC3-II/I ratio and beclin-1 level. These modifications help to enhance protective autophagy in HCC cells, thus inducing sorafenib resistance [82, 83]. Chen et al. reported that the combined use of hydroxychloroquine and sorafenib could overcome sorafenib resistance through inhibition of the Toll-like receptor 9 (TLR9)/SOD1/hsa-miR-30a-5p/beclin-1 resistance axis. Hsa-miR-30a-5p, a key miRNA targeting ATG5 and beclin-1, is significantly downregulated in drug-resistant cells; this alteration leads to significant autophagosome enrichment [84]. ATG4B plays a crucial role in autophagy and reportedly promotes HCC resistance [85]. Chen et al. found that the lncRNA CRNDE triggers protective autophagy by upregulating ATG4B in HCC cells via sequestration of miR-543, whereas sorafenib therapy activates the CRNDE/ATG4B/autophagy pathway and reduces sorafenib sensitivity in HCC [86]. Lin et al. found that METTL3 was significantly downregulated in sorafenib-resistant cells; its key downstream target was identified as FOXO3. METTL3 downregulation inhibits m6A-dependent FOXO3 methylation and directly induces the transcription of ATG3, ATG5, ATG7, ATG12, ATG16L1, and other ATGs in HCC. In addition, YTHDF1 is blocked from recognizing its m6A binding site, resulting in decreased FOXO3 mRNA stability; this process increases autophagic flux and induces sorafenib resistance [87]. Tao et al. found that the lncRNA BBOX1-AS1 was highly expressed in HCC. Furthermore, BBOX1-AS1 enhanced the stability of PHF8 mRNA by targeting miR-361-3p. PHF8 (i.e., KDM7B) is a histone demethylase that can trigger protective autophagy in HCC cells, leading to sorafenib resistance [53].

### Epigenetic modifications reprogram tumor metabolism

**Fig. 5:**
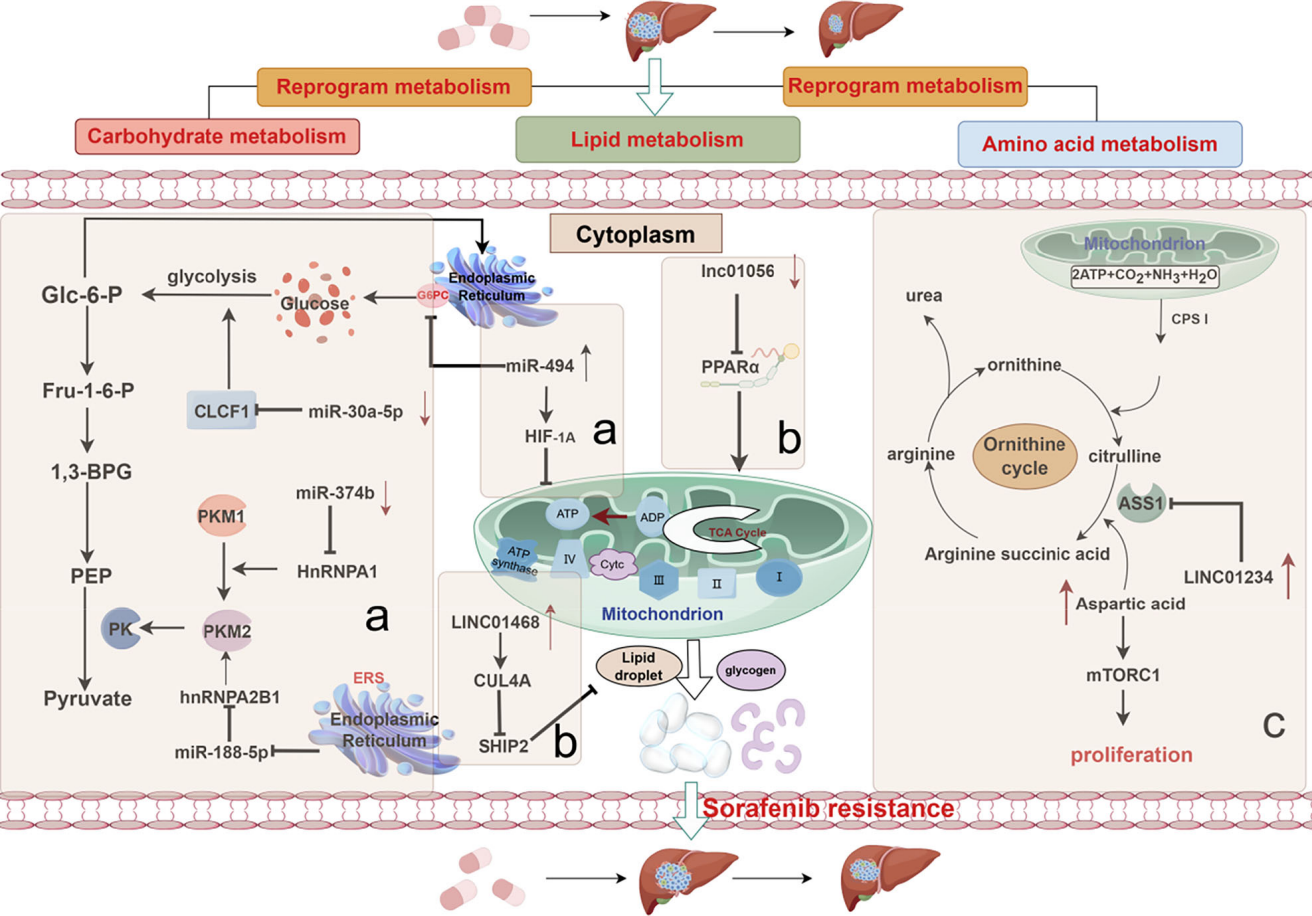
Epigenetic modifications promote sorafenib resistance through metabolic reprogramming, involving the following regulators. **a** miR-374b, miR-188-5p, miR-30a-5p, and miR-494 promote resistance by regulating glycolysis. **b** lncRNA LINC01468 and lncRNA LINC01056 promote resistance by regulating lipid metabolism. **c** lncRNA LINC01234 promotes resistance by regulating aspartate metabolism. Image created by Figdraw.

A defining feature of malignant tumors is unlimited proliferation, such that the body’s normal metabolism cannot meet the corresponding growth requirements. To solve this problem and adapt to the harsh conditions created by drug treatments, cancer cells rewire their metabolic pathways to ensure survival, proliferation, and metastasis. Unlike normal cells, which primarily rely on glucose metabolism to meet their energy needs, most malignant tumors harness glucose metabolism along with a range of other substances, including lipids and cholesterol, to produce the energy necessary for their unchecked growth. To maximize the utilization of these additional substances, cancer cells undergo autonomous epigenetic modifications that increase the expression of proteins associated with metabolic function, significantly reducing their susceptibility to targeted drugs and facilitating resistance [88, 89] (Fig. 5).

#### Carbohydrate metabolism

The liver has a high level of glucose metabolism, and aerobic glycolysis is essential for the rapid division of liver cancer cells. Zhang et al. found that glycolysis is usually more active in sorafenib-resistant HCC cells than in normal cells; this difference can be attributed to the downregulation of miR-374b expression in HCC cells under continuous sorafenib stimulation, which reduces the inhibitory effects on hnRNPA1. Upregulation of hnRNPA1 contributes to a shift in pyruvate kinase (PKM) gene splicing from PKM1 to PKM2. PKM2 is a key regulator of glycolysis and oxidative phosphorylation; its enrichment in rapidly proliferating cells leads to sorafenib resistance [90]. Another study showed that sorafenib resistance was mediated by endoplasmic reticulum stress (ERS) and subsequent PKM2 accumulation. ERS activation significantly downregulated miR-188-5p expression and directly upregulated hnRNPA2B1 expression in HCC cells. hnRNPA2B1 promotes survival and suppresses apoptosis by upregulating PKM2 [91]. Cardiotrophin-like cytokine 1 (CLCF1) is inversely regulated by upstream miR-30a-5p. Zhang et al. reported that sorafenib treatment significantly reduced the expression of miR-30a-5p, whereas overexpression of CLCF1 activated the phosphoinositide 3-kinase (PI3K)/AKT signaling pathway and multiple glycolytic genes. This enhanced glycolytic process produces sorafenib resistance in HCC cells [92]. High miR-494 levels negatively regulate glucose 6-phosphatase catalytic subunit (G6pc), resulting in the inhibition of glucose 6-phospho-glucose conversion; the accumulation of glucose 6-phosphate helps promote glycolytic metabolism and glycogen accumulation. In addition, miR-494 blocks oxidative phosphorylation by activating the hypoxia-inducing factor 1A (HIF-1A) pathway, which leads to lipid droplet storage. Enhancement of the glycolysis process and lipid droplet accumulation both create a favorable environment for HCC cell resistance to sorafenib-induced death, but the dominant role is glycolysis [93].

#### Lipid metabolism

Lipid metabolism reprogramming is a key feature of malignant tumors; the change of fatty acid metabolism in HCC cells is a distinguishing feature from normal liver cells. Increases in lipid uptake, storage, and lipogenesis contribute to rapid growth and drug resistance [94]. The prevalence of metabolic-associated fatty liver disease (MAFLD) is increasing worldwide. In MAFLD patients, the liver is overwhelmed by the need to degrade fatty acids, resulting in toxic lipid accumulation and an increased risk of liver cancer [95]. The lncRNA LINC01468, significantly upregulated in MAFLD and HCC, disrupts SH2 domain-containing inositol polyphosphate 5-phosphatase-2 (SHIP2) protein stability by modulating Cullin 4A (CUL4A)-mediated ubiquitination; this process activates the PI3K/AKT/mTOR signaling pathway, promoting de novo lipid biosynthesis and sorafenib resistance [96]. Chen et al. observed significantly downregulated expression of lncRNA LINC01056 in the context of sorafenib resistance, and the underlying mechanism involved cancer cell transformation from glycolysis to fatty acid oxidation. LINC01056 knockout cells showed high levels of fatty acid oxidation (FAO) activity during sorafenib treatment. HCC tumor cells preferentially use FAO (rather than glycolysis) to increase energy production (i.e., adenosine triphosphate [ATP] accumulation), yielding sorafenib resistance. Peroxisome proliferator-activated receptor α (PPARα) is a ligand-activated transcription factor responsible for maintaining a balance between FAO and glycolysis. LINC01056-mediated sorafenib resistance is achieved by regulating the post-transcriptional activity of PPARα [97]. From the above mechanisms, it is evident that the regulation of fatty acid oxidation (FAO), whether up- or downregulated, contributes to sorafenib resistance. Indeed, the emergence of drug resistance in cancers can be linked to energy accumulation, and FAO serves as a significant source of energy. When FAO levels decline, this is frequently accompanied by an intensification of glycolysis or an accumulation of lipid droplets. The accumulation of these lipid droplets allows HCC to switch to FAO in a timely manner to supply energy when glycolysis becomes insufficient. The metabolism of HCC is a highly intricate physiological process, with various metabolic pathways often not functioning independently but rather complementing each other. This interdependence may explain why the bidirectional regulation of FAO promotes sorafenib resistance.

#### Amino acid metabolism

Altered amino acid metabolism is needed to support the malignant tumor phenotype and enhance anticancer drug resistance. Aspartic acid is essential for protein synthesis and nucleotide biosynthesis; however, cancer cells cannot convert asparagine to aspartic acid because they lack asparaginase activity [98]. Therefore, cancer cells extensively reprogram intracellular aspartic acid cycling to maintain sufficient levels of aspartic acid. Chen et al. found that the lncRNA LINC01234 binds the promoter of arginine succinate synthase 1 (ASS1) to inhibit its transcriptional activation, thereby suppressing the conversion of aspartic acid to urea in cancer cells. In addition, the increase in cellular aspartic acid leads to activation of the mTORC1 pathway; increased mTORC1 activity similarly contributes to the development of sorafenib resistance in HCC [99].

### Epigenetic modifications facilitating the formation and maintenance of drug-resistant cells

**Fig. 6:**
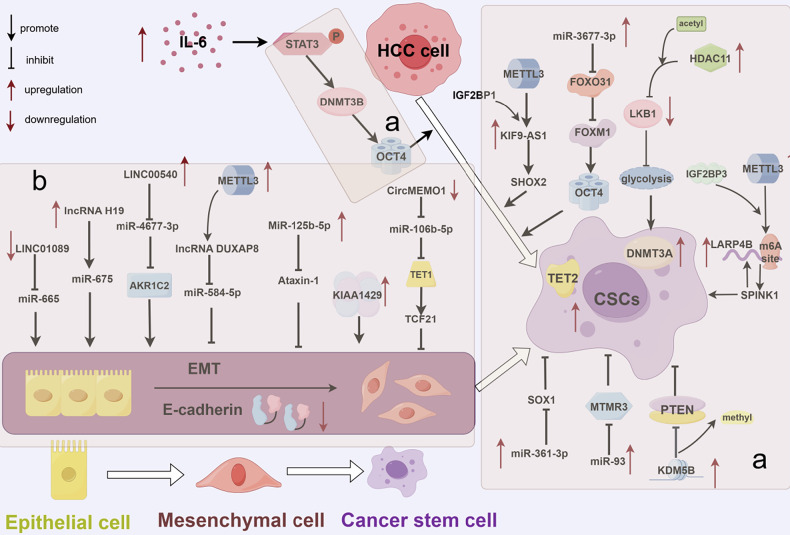
Epigenetic modifications promote sorafenib resistance by facilitating the formation and maintenance of drug-resistant cells, involving the following regulators. **a** miR-3677-3p, miR-93, miR-361-3p, DNMT3a, DNMT3b, TET2, KDM5B, HDAC11, and METTL3 drive resistance by stimulating CSC expansion. **b** LINC01089, lncRNA H19, miR-125b-5p, LINC00540, circMEMO1, lncRNA DUXAP8, KIAA1429, and METTL3 drive resistance through EMT activation. Image created by Figdraw.

Previous studies have demonstrated that stemness, which enables the formation and maintenance of drug-resistant cells, is a pivotal factor involved in drug resistance [100]. Stemness primarily refers to the phenomenon where, after the majority of cancer cells are eliminated by drug treatment, a small subpopulation survives and undergoes renewal. These surviving cells exhibit acquired drug resistance, which they can sustain and transmit over an extended period, thereby impeding tumor eradication [101] (Fig. 6).

#### Cancer stem cell expansion

Cancer stem cells (CSCs), alternatively referred to as tumor-initiating cells (T-ICs), constitute the primary cellular group that imparts tumor stemness [102]. These stationary, self-renewing cells reside within primary cancers, nestled in tumor niches where their extensive functional potential fuels cancer growth by re-establishing heterogeneity. Theoretically, a single CSC can rebuild an entire tumor; this near-immortality also provides robust treatment resistance [103].

MiR-3677-3p, upregulated in HBV + HCC, drives sorafenib resistance by suppressing F-box only protein 31 (FBXO31)—a tumor-suppressive stem cell factor (SCF) ubiquitin ligase component. FBXO31 downregulation reduces forkhead box protein M1 (FOXM1) ubiquitination, stabilizing this oncoprotein; FOXM1 further activates OCT4 to enhance CSC expansion, thus sustaining therapeutic resistance [104, 105]. Chen et al. also observed high expression of DNMT3a and TET2 in CSCs, revealing close coordination between these two enzymes. The DNMT3a–TET2 axis promotes oncosphere formation and enhances the tumor-reconstructing capacity of CSCs, thereby increasing HCC tolerance to sorafenib [106]. The upregulation of HDAC11 in HCC inhibits liver kinase B1 (LKB1) expression by triggering acetylation of histone H3 lysine 9 (H3K9ac) in the downstream LKB1 promoter region. Subsequent inhibition of the downstream AMPK signaling pathway indirectly enhances glycolysis and continuously activates CSCs, maintaining their stemness and sorafenib resistance [107]. MiR-93 is also highly expressed in liver T-ICs, where it enhances self-renewal and tumorigenesis by downregulating myotubularin-associated protein 3 (MTMR3) [108]. Qu et al. found that miR-361-3p upregulation promotes the expansion of liver T-ICs by negatively regulating SRY-box transcription factor 1 (SOX1) [109]. Furthermore, KDM5B (histone demethylase) is upregulated in sorafenib-resistant HCC and drives therapeutic resistance through activating PI3K/Akt pathway and CSC trait potentiation [110]. Lai et al. demonstrated that elevated serum IL-6 levels were associated with significantly shorter overall survival in HCC patients. IL-6 was shown to upregulate DNMT3b and OCT4 expression. As a critical transcription factor, OCT4 promotes the conversion of HCC cells into stem-like cells. Mechanistically, IL-6 enhances OCT4 expression via a STAT3-dependent pathway involving DNMT3b, potentially facilitating sorafenib resistance [111]. LARP4B, upregulated by METTL3-mediated, IGF2BP3-dependent m6A modification, promotes cancer stemness progression and impairs sorafenib efficacy by activating the SPINK1-mediated EGFR pathway. Inhibition of LARP4B enhances the antitumor effect of sorafenib by disrupting the positive feedback loop of the LARP4B/SPINK1/p-AKT/C/EBP-β axis [112]. METTL3 stabilized and increased lncRNA KIF9-AS1 expression through an m6A-IGF2BP1-dependent mechanism. KIF9-AS1 enhances cancer stemness and sorafenib resistance in HCC by promoting Ubiquitin-specific peptidase 1 (USP1)-mediated deubiquitination of short stature homeobox (SHOX2) [113].

#### Enhanced EMT

Epithelial–mesenchymal transition (EMT) is a process through which epithelial cells are transformed into mesenchymal cells. It is closely linked to the phenotypic heterogeneity of cancer cells and helps them rapidly adapt to various injuries, facilitating drug resistance [114]. Its key characteristics are loss of the epithelial marker E-cadherin and increased expression of the mesenchymal marker vimentin [115]. Notably, there exists a considerable overlap between the signaling pathways activated during EMT and those that propel cancer stem cells (CSCs), such as the Wnt, Hedgehog, and Notch signaling pathways. This overlap may offer partial insights into the heightened drug resistance [116]. Furthermore, certain epigenetic regulatory factors contribute to sorafenib resistance by regulating the expression of EMT-associated proteins.

Sun et al. detected significantly diminished expression of the lncRNA LIMT (LINC01089) in HCC, which was associated with poor prognosis. The downregulation of LINC01089 contributes to increased expression of miR-665, which promotes sorafenib resistance by enhancing EMT [117]. Xu et al. reported that the lncRNA H19 reduced E-cadherin expression by promoting the expression of miR-675 and enhancing tumor cell migration and invasion properties, leading to sorafenib resistance [118]. HCC cells with upregulated LINC00540 exhibited intrinsic sorafenib resistance, an irregular and elongated EMT-like phenotype, and decreased E-cadherin expression. This resistance mechanism is attributed to LINC00540-mediated upregulation of aldo-keto reductase family 1 member C2 (AKR1C2) expression and promotion of EMT through competitive binding with miR-4677-3p [119]. MiR-125b-5p also enhances EMT in HCC by targeting Ataxin-1; this interaction regulates sorafenib resistance [120]. Expression of the lncRNA DUXAP8 was significantly higher in HCC than in para-cancer normal tissues, potentially because of METTL3 binding to the m6A site on DUXAP8. Mechanistic analysis revealed that DUXAP8 activates the MAPK/ERK pathway via competitive binding to miR-584-5p, thereby enhancing globule formation by HCC cells and promoting the expression of stem cell-related genes [121]. KIAA1429, a core m6A methyltransferase component, is upregulated in sorafenib-resistant liver cancer cells. Ye et al. reported that aberrant upregulation of KIAA1429 and the associated m6A methylation led to enhanced EMT, partially explaining the intrinsic origin of sorafenib resistance [122].

CircMEMO1 functions as a molecular sponge for miR-106b-5p, enhancing TET1 expression and elevating Transcription Factor 21 (TCF21) mRNA 5hmC levels. This mechanism suppresses EMT and diminishes the stemness of HCC, thereby increasing the sensitivity of HCC cells to sorafenib. However, circMEMO1 expression is markedly downregulated in HCC tissues. The disruption of the circMEMO1/miR-106b-5p/TCF21 regulatory axis contributes to reduced sorafenib sensitivity in HCC [123].

### Epigenetic modifications activate the uncontrolled cell proliferation signaling pathway activity

**Fig. 7:**
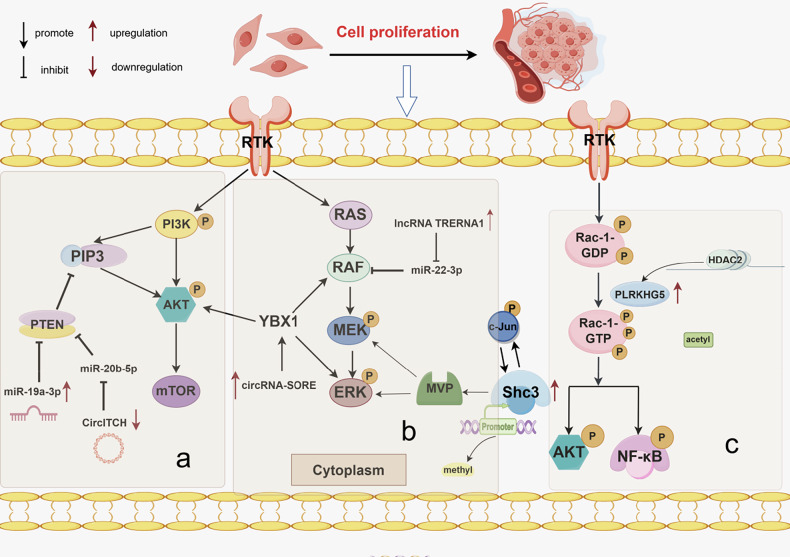
Epigenetic modifications promote sorafenib resistance by activating uncontrolled cell proliferation signaling pathways, involving the following regulators. **a** miR-19a-3p and circITCH drive resistance by modulating the PI3K/AKT/mTOR signaling pathway. **b** miR-22-3p, circRNA-SORE, and hypomethylation of the Shc3 upstream promoter drive resistance by modulating the Ras/Raf/MEK/ERK signaling pathway. **c** HDAC2 drives resistance by upregulating PLEKHG5 expression and activating the Rac1/AKT/NF-κB pathway. Image created by Figdraw.

Sorafenib primarily acts on serine-threonine kinases (e.g., Raf-1) and receptor tyrosine kinases such as VEGFR and platelet-derived growth factor receptor β (PDGFR-β). Complex signaling pathways within cancer cells respond to this targeted inhibition by suppressing angiogenesis and cell proliferation [124]. Although sorafenib influences metastasis and proliferation through multiple targets, such as VEGFR, Raf, and PDGFR, some HCC tumors do not overexpress these targets [125]. An additional challenge is that epigenetic changes also mitigate sorafenib toxicity by selectively downregulating important targets in cell signaling pathways. Here, we explore how sorafenib resistance is induced via dysregulation of downstream cell proliferation signaling pathways through epigenetic modifications (Fig. 7).

#### The PTEN/AKT/mTOR signaling pathway

The PI3K/AKT/mTOR signaling pathway is one of the most frequently dysregulated pathways in human cancers [126]. PTEN is an important tumor suppressor that antagonizes the highly carcinogenic AKT/mTOR pathway [127]. However, certain epigenetic regulators reduce PTEN expression or directly activate AKT, causing uncontrolled PTEN/AKT/mTOR signaling that desensitizes cancer cells to sorafenib [128]. For example, miR-19a-3p is highly expressed in HCC; it promotes HCC metastasis and sorafenib resistance by inhibiting PTEN [129]. CircITCH also participates in the downstream regulation of the PTEN/PI3K/Akt pathway by sponging miR-20b-5p, a negative regulator of PTEN. Continuous sorafenib treatment decreases circITCH expression in HCC cells and enhances downstream overexpression of miR-20b-5p, thereby reducing the tumor suppressor activity of PTEN [130].

#### The Ras/Raf/MEK/ERK signaling pathway

The Ras/Raf/MEK/ERK pathway is primarily known for promoting cell proliferation and survival, and its dysregulation is often associated with malignant tumor onset [131]. In addition, this pathway is involved in regulating apoptosis, cell cycle progression, cell migration, differentiation, metabolism, and other processes [132]. The significantly increased expression levels of Ras/Raf/MEK/ERK kinases in HCC play important roles in maintaining and promoting HCC differentiation [133]. Ras, a signal transduction activation switch, is reportedly mutated in 30% of HCC cases, suggesting that genetic alterations strongly contribute to HCC. Next, we discuss how the regulation of the Ras/Raf/MEK/ERK pathway activation by epigenetic modifications induces sorafenib resistance.

Positive expression of HBV-encoded X protein (HBx) is a poor prognostic factor for HCC patients. HBx induces upregulation of the lncRNA TRERNA1, which targets miR-22-3p to activate the Ras/Raf/MEK/ERK signaling pathway, thereby promoting cell proliferation and sorafenib resistance [134]. Xu et al. found that circRNA-SORE, upregulated in sorafenib-resistant HCC cells, hindered the degradation of Y-box-binding protein 1 (YBX1) by E3 ubiquitin ligase processing factor 19 (PRP19) and prevented nuclear translocation of the carcinogenic protein YBX1. Thus, overexpression of downstream targets, including AKT, Raf-1, and ERK, mediates sorafenib resistance [135]. Liu et al. found that Src collagen homology 3 (Shc3) was significantly upregulated in HCC compared with normal liver tissue; this upregulation was caused by hypomethylation of its upstream promoter, which enhanced c-Jun binding. The resulting positive feedback loop promoted high Shc3 expression and c-Jun phosphorylation. Using major vault protein (MVP) as a bridge, this positive feedback loop enhanced ShC3-mediated MEK and ERK phosphorylation, creating a resistance pathway independent of c-Raf [136]. In addition, Shc3 activates the β-catenin/T-cell factor (TCF) pathway by interacting with the cytoplasmic components of β-catenin, which reduces β-catenin degradation and increases the expression of MDR1 (multidrug resistance 1) in HCC, resulting in multidrug resistance [137].

#### Alternative signaling pathway

The aforementioned downstream pathways are classical downstream receptor tyrosine kinase (RTK) signaling pathways. Importantly, some HCC cells do not achieve resistance by direct inhibition of targeted drugs; instead, they activate alternative pathways that bypass the inhibitory effects of such drugs, promoting resistance. Sha et al. reported that sorafenib resistance can arise through activation of RTK replacement pathways to compensate for the loss of MAPK/ERK signaling pathways during sorafenib-mediated Raf inhibition. PLEKHG5, a Rho GTPase activator that responds to various extracellular and intracellular signaling pathways, is highly upregulated in sorafenib-resistant cells; HDAC2-mediated lysine deacetylation promotes its stability. PLEKHG5 overexpression promotes Rac1 and its downstream AKT/ NF-κB phosphorylation, reducing sorafenib sensitivity [138].

### Epigenetic modifications enable the abnormal transport process

**Fig. 8:**
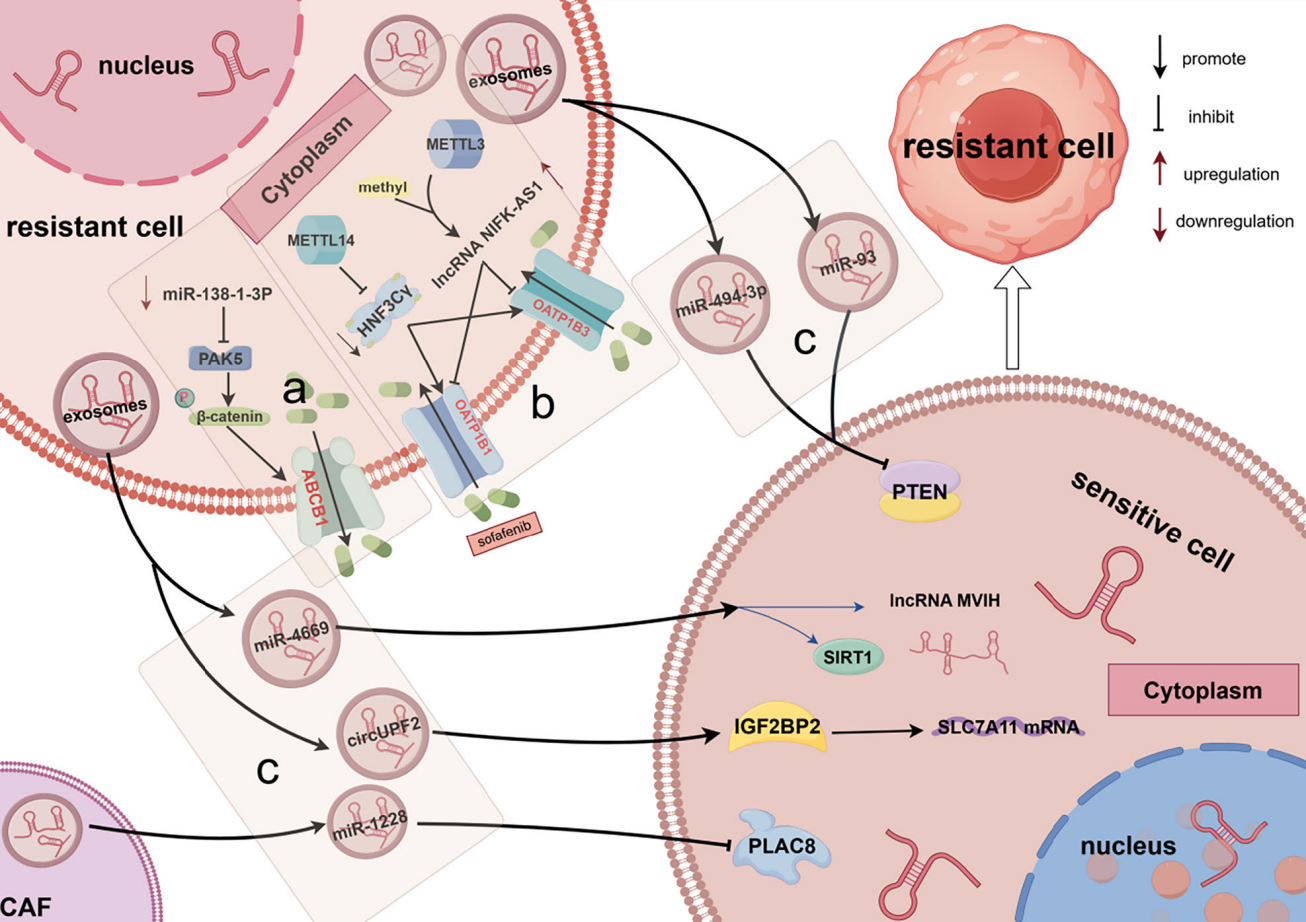
Epigenetic modification promotes sorafenib resistance by regulating the HCC transport process, involving the following regulators. **a** miR-138-1-3p promotes resistance by upregulating the drug efflux pump ABCB1. **b** METTL3 and METTL14 promote resistance by downregulating drug uptake transporters OATP1B1 and OATP1B3. **c** miR-4669, miR-1228, miR-494-3p, miR-93, and circUPF2 promote resistance via exosome-mediated transport. Image created by Figdraw.

The potency of the antitumor effects of targeted drugs partly depends on the concentration of the drug accumulated within tumor cells. In addition, the concentration of drugs within cancer cells hinges on the expression level of drug transporter proteins on the cell membrane. Malignant tumors can regulate the amount of transporter proteins through epigenetic mechanisms to decrease their internal drug concentration, thereby developing drug resistance. Another transport-related mechanism of drug resistance involves exosomes, which serve as crucial mediators for the transfer of drug resistance between resistant and sensitive cells, and are also regulated by epigenetic mechanisms. Here, we summarize the contributions of epigenetic modifications to transport-mediated sorafenib resistance in HCC (Fig. 8).

#### Drug transporters

The elevated expression of ATP-binding cassette (ABC) transporters in HCC promotes drug efflux–mediated drug resistance [139]. Some epigenetic regulators exacerbate sorafenib resistance by regulating ABC expression. For example, in the classical Wnt/β-catenin signaling pathway, β-catenin translocates into the nucleus to regulate gene expression activity. Li et al. reported that miR-138-1-3p was significantly downregulated in sorafenib-resistant HCC, accompanied by an upregulation of its target, p21-activated kinase 5 (PAK5). PAK5 enhances the phosphorylation and nuclear translocation of β-catenin, promoting the transcriptional activity of the drug efflux protein ABCB1. Increased ABCB1 expression promotes drug resistance by exporting sorafenib. Importantly, miR-138-1-3p mimics have the potential to reverse this resistance [140].

Another class of transporters has been identified; whereas ABC transporters are responsible for drug efflux, this new class of transporters is responsible for drug uptake into HCC cells [141]. Reduced expression of these transporters leads to resistance through insufficient drug accumulation within HCC cells [142]. Chen et al. reported that the lncRNA NIFK-AS1 is upregulated by METTL3-dependent m6A methylation in HCC, resulting in the downregulation of the drug transporters OATP1B1 and OATP1B3, which are mainly responsible for sorafenib uptake. This reduction in OATP1B1 and OATP1B3 levels impairs sorafenib sensitivity in HCC cells, thereby contributing to sorafenib resistance at high NIFK-AS1 levels [143]. Hepatocyte nuclear factor 3γ (HNF3Cγ) serves as the core of a regulatory system that promotes LCSC differentiation and inhibits malignant transformation. Importantly, it enhances the sorafenib response through activation of the drug transporters OATP1B1 and OATP1B3. However, Zhou et al. revealed that METTL14-dependent m6A modification downregulates HNF3C-γ expression, directly leading to sorafenib resistance [144].

#### Exosomes

Exosomes are cell-derived nanovesicles comprising bilateral lipid membranes that release their contents upon fusion with the cell membrane, facilitating intercellular communication and activating signaling pathways in target cells [145]. In addition, exosome contents usually include proteins, DNA, and ncRNAs; drug-sensitive cells can develop resistance by absorbing the contents of exosomes from drug-resistant cells [146]. HCC cells generally produce more exosomes than normal cells which contribute to sorafenib resistance in HCC cells.

High expression of miR-4669 in circulating exosomes is a risk factor for poor HCC prognosis. A study revealed that miR-4669-overexpressing HCC cells exhibited gradual upregulation of SIRT1 and elevated levels of the lncRNA MVIH. Although no direct interaction between SIRT1 and MVIH was evident, their overexpression promoted microvascular infiltration in HCC tissues and enhanced sorafenib resistance [147]. Extracellular vesicles (EVs) derived from cancer-associated fibroblasts (CAFs) are rich in miR-1228, which inhibits stress-induced apoptosis; the communication property of EVs helps to transmit this inhibition. CAF-derived EVs deliver miR-1228-3p, which enhances sorafenib resistance by modulating the placenta-related protein 8 (PLAC8)/PI3K/AKT axis [148]. The GOLPH3 oncoprotein is highly expressed in HCC, where it increases the BCL-2/Bax ratio and promotes sorafenib resistance by upregulating the exosomal level of miR-494-3p and enhancing PTEN targeting [149]. Huang et al. identified a significant enrichment of circRNA circUPF2 in exosomes derived from sorafenib-resistant HCC cells. Functioning as a molecular scaffold, circUPF2 recruits insulin-like growth factor 2 mRNA-binding protein 2 (IGF2BP2), which stabilizes the downstream target SLC7A11 mRNA. This stabilization suppresses ferroptosis in HCC cells, ultimately conferring sorafenib resistance to previously sensitive HCC cells [150]. MiR-93 is markedly overexpressed in sorafenib-resistant HCC cells, where it drives drug resistance by directly targeting PTEN. Notably, miR-93 is selectively packaged into exosomes secreted by these resistant cells. These miR-93-laden exosomes act as critical vehicles for disseminating sorafenib resistance, reactivating the PI3K/AKT signaling pathway through their PTEN-targeting activity [151].

## Epigenetic modifications involved in lenvatinib resistance

**Fig. 9:**
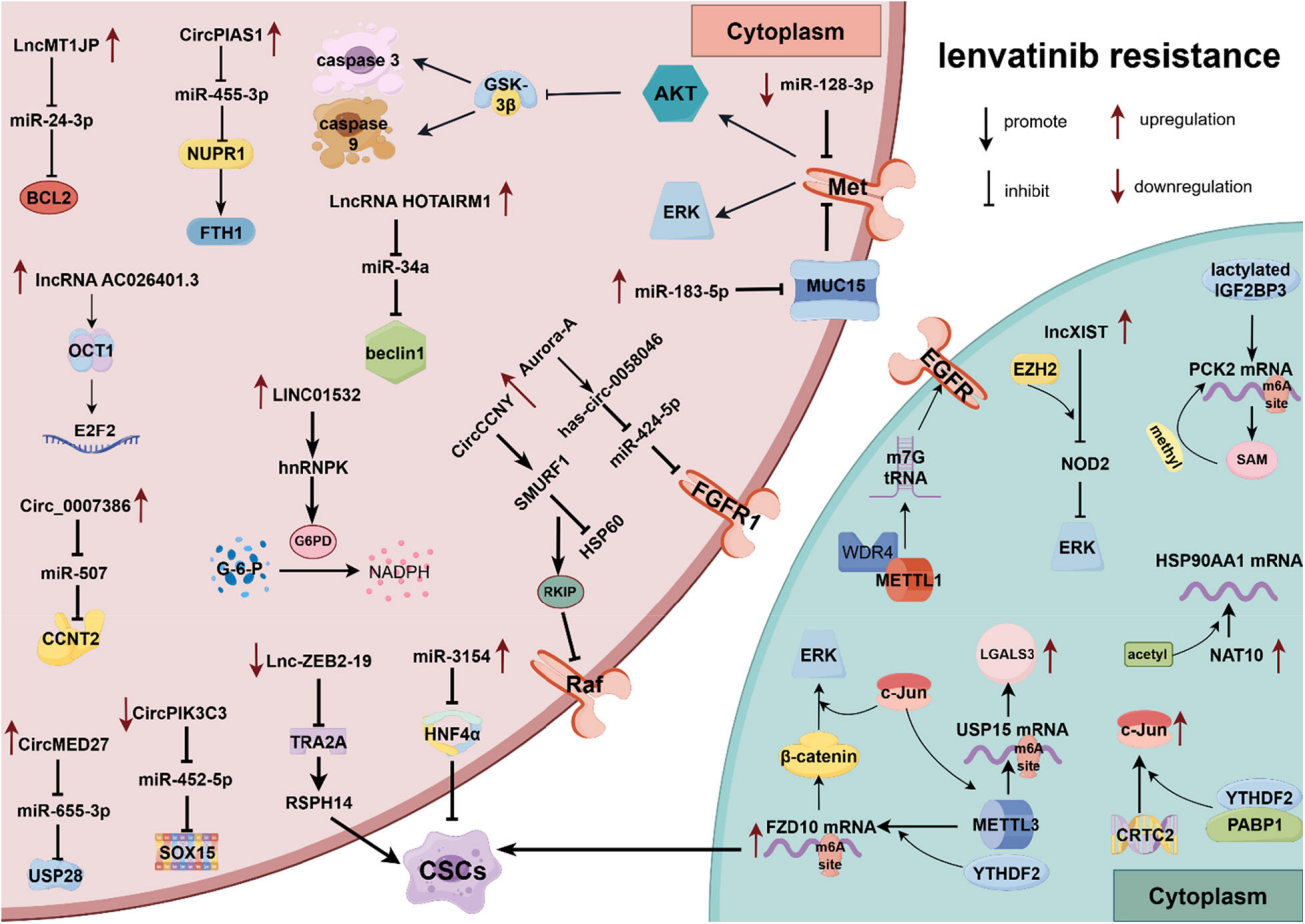
Epigenetic modifications play crucial roles in lenvatinib resistance. The reported epigenetic mechanisms of lenvatinib resistance involve ncRNA regulation, RNA methylation/acetylation, and histone modification. NcRNAs promotes lenvatinib resistance through regulating PCD (e.g., lncRNA AC026401.3, lncMT1JP, lncRNA HOTAIRM1, miR-128-3p and circPIAS1), CSCs expansion (e.g., miR-183-5p, miR-3154, LCC-ZEB2-19 and circ0007386), metabolism reprogramming (e.g., LINC01532), and uncontrolled cell proliferation signaling pathway activity (e.g., CircCCNY and hsa-circ-0058046); key regulators of RNA methylation/acetylation include METTL3, METTL1, YTHDF2, and NAT10; histone modifications involve IGF2BP3-mediated lactylation and EZH2-dependent NOD2 methylation. Image created by Figdraw.

More than a decade after the approval of sorafenib, a randomized phase III non-inferiority trial of lenvatinib demonstrated benefits in median and progression-free survival [152]. In 2018, lenvatinib was approved by the FDA as a first-line targeted therapy for unresectable HCC [153]. Lenvatinib has demonstrated non-inferiority to sorafenib in terms of overall survival (OS) and superiority across all secondary efficacy endpoints [154]. However, despite its promising efficacy, the treatment of HCC (HCC) with lenvatinib is similarly impeded by the emergence of drug resistance. Given its relatively shorter clinical application history compared to sorafenib, research on the mechanisms underlying lenvatinib resistance is somewhat limited. In this chapter, we present an overview of the reported epigenetic mechanisms involved in lenvatinib resistance, with a particular emphasis on ncRNA regulation, RNA methylation, and histone post-translational modifications (Fig. 9).

### NcRNA regulation promotes lenvatinib resistance

#### NcRNAs enables tumors to escape programmed cell death

Yu et al. showed that lenvatinib promoted the expression of lncMT1JP, which increased the downstream expression of BCL-2 via competitive binding to miR-24-3p and decreased the cleavage of caspase-3, thereby enhancing resistance to the apoptosis-inducing effect of lenvatinib [47]. Chronic lenvatinib exposure suppresses miR-128-3p expression in HCC cells, thereby driving hyperactivation of the c-Met/Akt/ERK signaling axis. Mechanistically, Akt activation inhibits phosphorylation of GSK3β, which subsequently blocks caspase-9 and caspase-3 proteolytic cleavage via GSK3β inactivation, ultimately establishing an anti-apoptotic program that confers lenvatinib resistance [155]. CircPIAS1 is upregulated in HCC and competitively sponges miR-455-3p to enhance NUPR1 expression, which transcriptionally activates FTH1. This cascade suppresses Fe²⁺-dependent Fenton reactions and confers lenvatinib resistance. Notably, combining ZZW-115 (a NUPR1 inhibitor) with lenvatinib synergistically suppresses tumor growth in circPIAS1-overexpressing murine models. Further investigation is warranted to delineate the precise mechanisms through which NUPR1 modulates ferroptosis pathways, which is critical for developing comprehensive therapeutic targeting strategies [156]. The lncRNA HOTAIRM1, a conserved Hox gene cluster, is significantly upregulated in lenvatinib-resistant HCC; it activates the downstream autophagy-related factor beclin-1 and triggers autophagy to promote lenvatinib resistance through the suppression of miR-34a, a molecular sponge of HOTAIRM1 [157].

#### NcRNAs enable the formation and maintenance of drug-resistant cells

Han et al. demonstrated that miR-183-5p overexpression in HCC induces MUC15 downregulation. MUC15 can restrict T-IC expansion by destabilizing c-MET and suppressing PI3K/AKT/SOX2 signaling. MUC15 degradation led to an increased proportion of T-ICs in HCC tissues, enhancing malignant proliferation and promoting lenvatinib resistance [158]. In addition, HCC cells and LCSCs display elevated expression of miR-3154, which promotes self-renewal in HCC by downregulating the mRNA and protein expression levels of hepatic nuclear factor 4 alpha (HNF4α). This effect of miR-3154 results in a poor response to lenvatinib [159]. Lnc-ZEB2-19 suppresses HCC stemness and metastasis by binding to Transformer 2α (TRA2A) to induce RSPH14 mRNA degradation. Paradoxically, the expression of lnc-ZEB2-19 is commonly downregulated in HCC, which promotes lenvatinib resistance development [160]. Circ0007386 is significantly upregulated in HCC and acts as a molecular sponge for miR-507 to alleviate its suppression of CCNT2, thereby driving EMT and lenvatinib resistance [161].

#### NcRNAs reprogram tumor metabolism

LINC01532 exhibits m6A-mediated upregulation in HCC and interacts with hnRNPK to facilitate G6PD pre-mRNA splicing. This post-transcriptional regulation enhances G6PD expression and pentose phosphate pathway (PPP) flux, resulting in accelerated NADPH biosynthesis. The accumulated NADPH reinforces cellular antioxidant defenses, thereby attenuating lenvatinib-induced oxidative stress and apoptosis in HCC cells [162].

#### NcRNAs enable uncontrolled cell proliferation signaling pathway activity

CircCCNY recruits the E3 ubiquitin ligase SMURF1 to induce HSP60 ubiquitination and proteasomal degradation. This process liberates Raf kinase inhibitory protein (RKIP), thereby inactivating the MAPK survival signaling pathway through RKIP-mediated phosphatase recruitment, which consequently potentiates lenvatinib-induced tumor cell apoptosis. However, high-throughput sequencing analysis revealed a paradoxical downregulation of circCCNY in HCC, which likely impairs circCCNY’s ability to suppress the MAPK pathway, thereby contributing to lenvatinib resistance [163]. HCC with high basal Aurora-A expression levels is more prone to developing lenvatinib resistance. The underlying mechanism involves Aurora-A positively regulating hsa-circ-0058046 to competitively inhibit miR-424-5p and activate FGFR1 signaling. Combination therapy using the Aurora-A inhibitor MLN-8237 with lenvatinib synergistically reverses lenvatinib resistance. However, this study lacked human tissue samples to measure hsa-circ-0058046 expression in cancerous tissues and analyze its correlation with clinical features, particularly regarding lenvatinib resistance [164].

### RNA methylation promotes lenvatinib resistance

METTL3 catalyzes m6A methylation of frizzled10 (FZD10) mRNA to modulate FZD10 expression. YTHDF2 is responsible for stabilizing the modified FZD10 mRNA. After upregulation, FZD10 promotes self-renewal of hepatic CSCs by activating β-catenin and YAP1; it also reduces HCC cell sensitivity to lenvatinib by activating the β-catenin/c-Jun/MEK/ERK axis. Furthermore, the promoter region of METTL3 contains c-Jun binding sites that enhance METTL3 expression, forming a stable positive feedback loop that contributes to persistent lenvatinib resistance [165]. In addition, mettl3-mediated m6A modification upregulates USP15 expression in HCC. USP15 stabilizes Lectin Galactoside-Binding Soluble 3 (LGALS3) by blocking its ubiquitin-proteasome system-mediated degradation, thereby activating the AKT/mTOR axis to promote cancer stemness, proliferation, and lenvatinib resistance. However, this study lacks clinical validation of the USP15/LGALS3/AKT/mTOR axis in lenvatinib-treated patient specimens, and the mechanistic details underlying USP15-driven AKT/mTOR activation remain unresolved [166]. METTL1 and WD repeat-containing protein 4 (WDR4) were significantly upregulated in lenvatinib-resistant cells. METTL1/WDR4 complex promoted the translation of EGFR pathway proteins through tRNA N7-methylguanosine (m7G) methylation modification, thereby conferring lenvatinib resistance [167]. Wang et al. identified an amplification of the transcriptional coactivator 2 (CRTC2) gene located in the 1q21.3 region. CRTC2 interacts with PABP1 and recruits YTHDF2 to enhance the translation of specific m6A-mRNA via relocating mRNA from decay sites to polysomes. CRTC2-mediated enhanced translation of c-Jun confers resistance to lenvatinib [168].

N4-acetylcytidine (ac4C) is a conserved acetylation modification of mRNA. Although less studied than m6A, ac4C plays an important role in regulating gene expression stability [169]. For example, ERS is a cancer cell defense mechanism induced by extracellular and intracellular stresses; this mechanism directly enhances the invasiveness and metastasis abilities of HCC cells by modifying the tumor microenvironment [170]. Pan et al. discovered that NAT10 is a lysine acetyltransferase that enhances ac4C modification of HSP90AA1 RNA and increases lenvatinib resistance in HCC cells during ERS [171].

### Histone modification promotes lenvatinib resistance

Duan et al. reported that lncXIST was significantly upregulated in HCC and bound to the histone-modifying enzyme EZH2. This interaction led to increased H3K27me3 in the promoter region of nucleotide-binding oligomerization domain 2 (NOD2), thereby inhibiting NOD2 gene expression. Downregulation of the tumor suppressor NOD2 and subsequent activation of downstream ERK targets may partially explain the development of lenvatinib resistance [172, 173]. Lu et al. discovered that HCC exhibit elevated lactylation levels, which promote lactylation at the K76 site of insulin-like growth factor 2 mRNA-binding protein 3 (IGF2BP3), enhancing its binding to m6A-modified phosphate carboxykinase 2 (PCK2) and nuclear factor erythroid 2-related factor 2 (NRF2) mRNAs and boosting the antioxidant capacity of HCC cells. Upregulated PCK2 redirects carbon flux toward serine metabolism and one-carbon unit synthesis, maintaining high levels of S-adenosylmethionine (SAM) and serine. This metabolic shift reinforces m6A modification of PCK2 and NRF2 mRNAs, creating a positive feedback loop of IGF2BP3 lactylation-PCK2-SAM-m6A. This loop sustains elevated PCK2 and NRF2 expression, ultimately driving lenvatinib resistance [174].

### Other lenvatinib mechanisms

CircMED27 exhibited marked upregulation in both serum and tumor tissues of HCC patients. circMED27 functions as a molecular sponge for miR-655-3p, effectively relieving its inhibitory effect on the target gene USP28, thereby promoting USP28 protein expression. Subsequent functional analyses confirmed that this circMED27/miR-655-3p/USP28 regulatory axis critically contributes to the development of lenvatinib resistance in HCC [11]. CircPIK3C3 is significantly downregulated in HCC, which relieves its negative regulation of miR-452-5p, leading to activation of the Wnt/β-catenin signaling pathway and downregulation of the downstream tumor suppressor gene SOX15. This process has been demonstrated to be closely associated with lenvatinib resistance. However, the study did not establish a direct or more comprehensive link between SOX15 and lenvatinib resistance [175].

### Shared epigenetic resistance mechanisms/targets of sorafenib and lenvatinib

The epigenetic mechanisms underlying sorafenib/lenvatinib resistance encompass shared regulatory processes, including DNA/RNA methylation, histone modifications, and non-coding RNA regulation, and shared resistance outcomes involving dysregulated programmed cell death, metabolic reprogramming, enhanced cancer stem cell properties, and uncontrolled proliferative signaling pathways. Moreover, they exhibit shared core mechanisms in drug resistance evolution.

LncRNA Nuclear Paraspeckle Assembly Transcript 1 Variant 1 (NEAT1v1) switches HCC growth pattern from MEK/ERK-dependent to AKT-dependent via superoxide dismutase 2 (SOD2), conferring resistance to both sorafenib and lenvatinib [176]. HCC cells with upregulated lncRNA AC026401.3 expression exhibit lenvatinib/sorafenib resistance. Mechanistically, AC026401.3 facilitates OCT1 recruitment to the E2F2 promoter, promoting its transcription. This transcriptional activation establishes an imbalance wherein tumor proliferation outpaces drug-induced apoptosis, ultimately driving resistance during prolonged exposure [177]. Overexpressed YTHDF1 in CSCs drives CSC renewal and directly interacts with m6A-modified NOTCH1 transcripts, stabilizing their expression to promote lenvatinib/sorafenib resistance, exhibiting a dose-dependent resistance profile. Therapeutically, LNP-encapsulated YTHDF1-targeting siRNA synergistically suppressed tumor progression when combined with lenvatinib/sorafenib in murine models, revealing translational potential [178]. Arechederra et al. identified an association between ADAMTSL5 hypermethylation and TKI resistance. Mechanistically, ADAMTSL5 enhanced resistance to multiple TKIs (e.g., sorafenib, lenvatinib) through RTK signaling activation, without altering receptor protein levels [179].

In addition, sorafenib and lenvatinib resistance mechanisms share common molecular targets. For instance, the LARP4B/IGF2BP3/SPINK1 axis mediates sorafenib resistance, while the IGF2BP3 lactylation-PCK2-SAM-m6A axis drives lenvatinib resistance, with IGF2BP3 serving as a shared target in both pathways.

## Epigenetic drug resistance therapy and clinical translation

### Targeting strategies for epigenetic drug resistance

Previous sections have outlined epigenetic mechanisms (e.g., DNA/RNA methylation, histone/non-coding RNA modifications) driving sorafenib/lenvatinib resistance in HCC. The central challenge lies in targeting these pathways to overcome resistance and guide clinical translation strategies. The epigenetic alterations in drug-resistant HCC cells offer novel avenues for early detection and modulation of drug resistance. The comprehensive review of epigenetic therapies provides valuable insights for developing strategies to reverse drug resistance and advancing their clinical translation.

#### Epigenetic drugs

Epigenetic therapies highlight the potential of epigenetic drugs to reverse HCC resistance. Current strategies focus on two approaches: the first approach involves combination therapies with other anticancer agents to block or reverse established drug resistance after its emergence [180]. The alternative strategy focuses on monitoring epigenetic changes during treatment and intervening with epigenetic drugs before the stabilization of resistant epigenetic states, which may delay or prevent resistance development—potentially enabling cancer to be managed as a chronic condition [181]. The two most extensively studied classes of epigenetic drugs currently are DNA methyltransferase inhibitors (DNMTis) and histone deacetylase inhibitors (HDACis) [182].

DNMTis, such as FDA-approved 5-azacytidine (5-aza) and decitabine (DAC), reverse malignant phenotypes by reactivating tumor suppressor genes via DNA hypomethylation. Other DNMTis that are not yet approved include SGI-110 (guadecitabine) and the recently developed selective DNMT1 inhibitor GSK3685032183 [183, 184]. To address the short half-life of 5-aza and DAC, zebularine, next-generation DNMTi, is an orally administered and more stable DNMTi that has been demonstrated to have preclinical efficacy [185]. Emerging strategies combining DNMTis with sorafenib/lenvatinib demonstrate potential to reverse HCC resistance. For instance, nanaomycin A, a streptomyces-derived DNMT3b-selective quinone antibiotic, synergizes with sorafenib by suppressing IL-6-induced DNMT3b/OCT4 expression and restoring sorafenib sensitivity [111].

HDACis include vorinostat, belinostat, panobinostat, and romidepsin (FDA-approved), alongside investigational agents such as givinostat, resminostat, abexinostat, entinostat, mocetinostat, valproic acid, and butyrate [184, 186]. Combining HDACis with sorafenib/lenvatinib shows promise in alleviating sorafenib/lenvatinib resistance. For instance, a phase I/II trial combining the HDAC inhibitor resminostat with sorafenib demonstrated safety and tolerability, achieving a 90% disease control rate in sorafenib-refractory HCC patients [187]. The HDACi vorinostat reverses lenvatinib resistance by suppressing AKT activation, while co-treatment with lenvatinib and AKT/HDACi synergistically induces HCC cell apoptosis [188]. Additionally, EZH2, as a key regulator of histone methylation, can be targeted by drugs that inhibit its activity to modulate epigenetic processes. Currently, the approved EZH2 inhibitors include tazemetostat and valemetostat (approved only in Japan), while other drugs in clinical trials include GSK126, CPI-1205, SHR2554, and PF-06821497 [189]. Studies have reported on the combined use of EZH2 inhibitors with targeted therapies to overcome drug resistance. Tazemetostat synergizes with sorafenib to enhance ferroptosis in sorafenib-resistant HepG2-SR cells [74].

Currently, epigenetic drugs remain limited to a few FDA-approved agents, with most candidates in preclinical or early clinical development. Future research should prioritize validating their therapeutic efficacy in HCC to unlock their significant potential. The synergistic combination of epigenetic drugs with sorafenib/lenvatinib poses a significant threat to resistant tumors. Despite substantial challenges in clinical translation, advances in research and technology are expected to accelerate their therapeutic implementation.

#### Targeting NcRNAs

NcRNAs are increasingly recognized as pivotal epigenetic regulators of cancer therapy resistance. Targeting these ncRNAs holds promise for reversing drug resistance and advancing clinical strategies. Current approaches primarily involve silencing oncogenic ncRNAs via small interfering RNA (siRNA)/short hairpin RNA (shRNA) -mediated knockdown. For example, lncRNA SNHG1 depletion restores sorafenib-induced apoptosis in HCC by suppressing the miR-21/SLC3A2/AKT axis [79]. Similarly, circRNA-SORE silencing enhances sorafenib sensitivity through YBX1 downregulation [135]. However, this paradigm does not universally apply, as resistance can also be mediated by downregulation of tumor-suppressive ncRNAs. Restoring their expression via mimics may resensitize resistant cells to therapy. For instance, transfection of miR-128-3p mimics into HCC cells rescues lenvatinib’s anti-proliferative efficacy in resistant HCC models [155]. The combination of specific inhibitors of ncRNAs with targeted drugs has also demonstrated promising efficacy against drug-resistant tumors. The combination of lncRNA MALAT1-IN1 (MALAT1 inhibitor) with sorafenib demonstrated enhanced antitumor efficacy in preclinical HCC models [62].

While some strategies have been validated in preclinical models, others await rigorous exploration. Integrating mature gene-editing technologies with clinical regimens may unlock novel solutions to combat resistance.

#### Targeting m6A modification

Strategies to reverse HCC resistance by regulating the m6A process have become a research hotspot in recent years. For instance, METTL3 enhances EGFR mRNA stability via m6A modification, promoting lenvatinib resistance. However, the METTL3 inhibitor STM2457 improved tumor response to lenvatinib in multiple murine HCC models [190]. In addition, WD6305, as a potent and selective proteolysis-targeting chimera (PROTAC) degrader of the METTL3-METTL14 complex, also offers a novel approach for the treatment of drug-resistant tumors [191]. Notably, studies have reported that YTHDC1 ablation induces nuclear accumulation of FTH1/FTL transcripts, thereby partially counteracting sorafenib resistance [75].

Accumulating evidence indicates that natural products can reverse drug resistance by suppressing m6A modification. Liu et al. demonstrated that the natural compound rabdosiin effectively counteracts METTL8-mediated lenvatinib resistance [192]. Zhao et al. reported that lobeline, a plant-derived natural product, reverses lenvatinib resistance in HCC through m6A modulation, potentially via UBE3B-related mechanisms [193]. RNA methylation is a relatively newer form of epigenetic regulation, and in the future, more methylation inhibitors with targeted catalytic activity are expected to be developed.

#### Other epigenetic therapies

Certain therapeutic strategies do not directly target epigenetic regulators but instead act on upstream or downstream molecules of these regulatory molecules to indirectly reverse drug resistance, independent of epigenetic drugs or genetic interference. For example, the synergistic treatment with PUFA supplementation and sorafenib can overcome HNF4A-AS1/DECR1/PUFA axis-induced sorafenib resistance [68]. The glycolysis inhibitor 2-DG suppresses IGF2BP3 accumulation and its lysine lactylation, thereby reversing IGF2BP3 lactylation-mediated lenvatinib resistance [174]. Furthermore, some physiological substances have been shown to reverse drug resistance by modulating DNA methylation status. For instance, human menstrual blood-derived stem cells (MenSCs) enhance TET2 expression to reverse BCL-2-interacting protein 3 (BNIP3)/ BCL-2-interacting protein 3-like (BNIP3L) promoter hypermethylation in sorafenib-resistant HCC, thereby amplifying sorafenib-induced mitophagy and resistant-cell death [194].

The fundamental efficacy of these therapies stems from disrupting any component within the epigenetic drug resistance axis, thereby countering drug resistance. This implies that a comprehensive exploration of targets in resistance mechanisms could expand therapeutic options for epigenetic treatments. However, compared to therapies directly targeting epigenetic regulatory molecules, research on their efficacy remains more limited.

### Epigenetic therapies and types of drug resistance

The resistance to targeted therapy in HCC stems from either primary resistance or acquired resistance. Primary resistance typically involves inherent genetic alterations in tumors, manifesting as significant drug resistance during initial targeted treatment [195, 196]. For instance, elevated intrinsic Aurora-A expression predisposes HCC to lenvatinib resistance [164]. Moreover, genomic amplification of CRTC2 (1q21.3) promotes intrinsic lenvatinib resistance via co-condensate formation with the m6A reader YTHDF2, representing a novel innate resistance mechanism in HCC [168, 197]. In contrast, acquired resistance develops through dynamic adaptive mechanisms during treatment, enabling certain tumor cells to survive therapy and evolve new resistant phenotypes. Clinically, this manifests as a gradual loss of therapeutic efficacy after an initial response period [198]. A representative example is the cDCBLD2/miR-345-5p/TOP2A resistance regulatory mechanism reported by Ruan et al. through in vitro establishment of sorafenib-resistant cell lines [53]. Importantly, most of the drug resistance mechanisms summarized in this review fall into the category of acquired drug resistance. A hallmark feature of this resistance is its reversible nature, with drug sensitivity recoverable through genetic or pharmacological targeting of resistance-associated regulatory factors.

Fundamentally, primary resistance originates from tumor’s inherent stable state, while acquired resistance represents a dynamic and reversible adaptive response. Generally, the prognosis of patients with primary resistance is worse than that of those with acquired resistance [199]. The significance of distinguishing between the two types of resistance for epigenetic therapy lies in selecting the appropriate treatment timing. The optimal intervention window for acquired drug resistance should target the early stages of resistance evolution. By monitoring dynamic changes in drug resistance regulatory factors and implementing epigenetic-targeted therapy during the incipient phase of resistance development, when tumors have not yet established a stable resistance ecosystem and residual sensitive cells remain responsive to the original treatment regimen, it becomes possible to effectively prevent further progression of drug resistance [200–202]. However, overcoming intrinsic resistance necessitates early epigenetic/genetic interventions, provided that pretreatment detection of drug resistance-associated biomarkers could potentially prevent unnecessary delays in patient treatment caused by primary resistance. Regrettably, the prediction of potential drug resistance factors faces multiple obstacles in terms of cost, testing time, sensitivity, and specificity [203].

### Epigenetic therapy and HCC heterogeneity

HCC displays marked therapeutic heterogeneity across etiological subgroups. Comparative clinical evidence demonstrates lenvatinib’s superior efficacy over sorafenib in HBV-associated HCC, while showing comparable outcomes in HCV-positive patients [204]. Intriguingly, sorafenib exhibits enhanced therapeutic responses in HCV-related HCC [205]. Differences in therapeutic responses due to etiological variations are also observed in epigenetic drug resistance regulation. HBV-associated HCC shows significant miR-193b downregulation, driving sorafenib resistance through myeloid cell leukemia-1 (Mcl-1) overexpression [206], whereas HCV-related HCC exhibits miR-193b upregulation and Mcl-1 downregulation, which enhances therapeutic sorafenib sensitivity [207]. Lai et al. reported that IL-6 promotes sorafenib resistance through the DNMT3b-OCT4-DNMT1 axis. Targeting DNMT3b enhances sorafenib sensitivity and improves its therapeutic efficacy against sorafenib-resistant HCC cells, particularly in HBV^+^ HCC [111] findings demonstrate that epigenetic drug resistance mechanisms and therapeutic strategies in HCC exhibit significant heterogeneity depending on etiological drivers. Characterizing these etiology-driven epigenetic divergences is critical for guiding precision therapeutic strategies.

Emerging evidence indicates that hepatitis B virus X protein (HBx) promotes HBV-HCC development and progression through interactions with DNMT3A, HDAC1, and ncRNAs [208, 209]. In addition, studies have reported HBx-mediated epigenetic mechanisms contributing to drug resistance. For example, the aforementioned TRERNA1/miR-22-3p/NRAS axis sustains RAS/MAPK pathway activation, conferring sorafenib resistance in HCC cells. MiR-3677-3p suppresses the expression of FBXO31, thereby promoting sorafenib resistance in HBV-HCC [104]. Whether these drug resistance mechanisms identified in HBV-HCC are similarly present in HCV-HCC, and whether significant differences exist between the two etiologies, warrants systematic investigation.

The escalating global burden of type 2 diabetes and obesity has established MAFLD as a predominant HCC etiology [210]. Clinical analyses indicate that MAFLD-associated HCC patients typically present with older age at diagnosis, larger tumor dimensions, and more advanced disease stages. Notably, sorafenib demonstrates comparable therapeutic efficacy in MAFLD-related HCC to other etiological subtypes [211], and this finding also applies to lenvatinib [212]. Although Tomonari et al. reported enhanced responsiveness of lenvatinib in non-viral HCC compared to viral hepatitis-associated cases, these findings remain preliminary due to the small cohort size and restriction to Child-Pugh class A patients. Nevertheless, this observation underscores the importance of etiological considerations in therapeutic decision-making [213]. Consequently, elucidating epigenetic resistance mechanisms in MAFLD-related HCC remains imperative. Current mechanistic insights remain sparse, though pivotal findings emerge: Wang et al. demonstrated a mechanistic linkage between LINC01468 upregulation and SHIP2 destabilization via CUL4A-dependent ubiquitination in MAFLD-HCC. This LINC01468/SHIP2 axis drives PI3K/AKT/mTOR pathway activation, promoting sorafenib resistance [96]. Critical knowledge gaps persist, demanding systematic exploration of MAFLD-specific resistance networks and cross-etiological comparisons of their activation patterns to inform etiology-adapted therapeutic strategies.

Systematic comparative analysis of these etiological differences holds transformative potential for developing context-specific therapeutics, enabling rational therapeutic design to overcome clinical drug resistance through precision oncology frameworks in the future.

### Epigenetic therapy and the complexity of drug resistance

#### Bidirectional regulation of epigenetic molecules

The effects of epigenetic regulatory factors on downstream targets are not static. The same regulatory element can have completely opposite regulatory effects on different downstream molecules. This makes drug resistance in epigenetic regulation even more unpredictable and simultaneously highlights the necessity of identifying cross-targets involved in multiple drug resistance mechanisms. YTHDF2 specifically binds to m6A-modified mRNAs through its YTH domain, typically mediating target mRNA degradation or translational repression. For instance, CRTC2 activity counteracts YTHDF2-mediated mRNA degradation, thereby promoting lenvatinib resistance [168]. Beyond its degradation function, YTHDF2 paradoxically promotes drug resistance through mRNA stabilization mechanisms. YTHDF2 stabilizes m6A-modified FZD10 mRNA to promote lenvatinib resistance [165]. METTL3- and METTL14-mediated m6A modification of SREBF2-AS1 promotes HCC progression and sorafenib resistance [214], while METTL14-dependent m6A methylation similarly induces sorafenib resistance through downregulation of HNF3γ mRNA [144]. Similar scenarios exist in histone modifications. For instance, H3K27me3 serves as a repressive mark, whereas H3K4me is found at the promoters of active genes [30].

#### Epigenetic therapy and drug resistance networks

Emerging studies have demonstrated intricate interactions among DNA/RNA methylation, histone modifications, and ncRNA regulation [215], constructing a sophisticated regulatory network underlying sorafenib/lenvatinib resistance. This network manifests as cooperative crosstalk between diverse epigenetic regulators, which collectively drive drug resistance through multilayered molecular adaptations. For instance, m6A-modified SREBF2-AS1 orchestrates TET1 and FXR1 recruitment to activate SREBF2 transcription in sorafenib-resistant HCC [214]. CircMEMO1 modulates sorafenib therapeutic sensitivity by regulating the promoter methylation status of TCF21 [123]. Similarly, lenvatinib resistance involves lncXIST-driven EZH2/H3K27me3 axis activation [177] and IGF2BP3 K76 lactylation-PCK2 m6A cooperation, enhancing antioxidant defenses [174]. MiR-21 promotes the methylation of PTEN by regulating the expression of the TET protein family, thereby enhancing HCC resistance [216].

The intricate drug resistance regulatory network poses a further crisis for epigenetic therapy, as monotherapy with a single epigenetic drug demonstrates poor efficacy in overcoming drug resistance that involves multiple regulatory mechanisms. Combination therapy with multiple epigenetic drugs demonstrates therapeutic promise: DNMTi-induced DNA demethylation fails to reactivate tumor suppressor genes due to persistent PRC2-mediated repression. As EZH2 catalyzes PRC2-dependent H3K27 trimethylation, combining EZH2i with DNMTis synergistically reactivates epigenetically silenced therapeutic targets. Preclinical validation demonstrated DNMTi/EZH2i co-treatment (DAC + GSK126) enhances antitumor immunity and suppresses cancer stemness markers in HCC models, outperforming DNMTi monotherapy [217]. While limited to preliminary cell line data, these findings underscore the necessity of multi-mechanism targeting to counteract epigenetic networks.

### Personalized epigenetic therapy

The genetic basis for implementing personalized treatment is the heterogeneity of HCC. Essentially, tumor heterogeneity determines the different epigenetic drug resistance mechanisms among patients. Personalized epigenetic therapy embodies a precision oncology paradigm tailoring therapeutic regimens to individual epigenetic differences, aiming to optimize therapeutic outcomes while mitigating off-target toxicity. Central to this approach is the systematic identification of tumorigenic epigenetic drivers coupled with the application of modality-specific epigenetic modifiers or genome-editing technologies [218]. For instance, ncRNAs expression in drug-resistant tumors can be either upregulated or downregulated, depending on the specific ncRNAs ‘ role in the resistance mechanism. Clinically, these differentially expressed miRNAs emerge as actionable biomarkers detectable during incipient resistance phases, enabling preemptive therapeutic adjustments [219].

The clinical efficacy of epigenetic agents demonstrates interpatient variability attributable to tumor heterogeneity. This therapeutic challenge necessitates precision selection of targeted epigenetic modulators based on individual resistance mechanisms to achieve personalized therapeutic suppression of drug-resistant pathways. For instance, while histone modification-driven resistance mechanisms may share common epigenetic foundations, their distinct molecular mediators require tailored pharmacological interventions to optimize therapeutic outcomes. As evidenced by our prior investigations, histone demethylases KDM1A and KDM5B, respectively, drive drug resistance, wherein targeted pharmacological inhibition using respective antagonists effectively reverses these resistance mechanisms. For example, the combination of a KDM5B inhibitor and lenvatinib can restore p15 levels and enhance the sensitivity of resistant cells to lenvatinib [220]. KDM5B-targeting drugs include: CPI-455, KDM5-C49, and GSK-J1 [221]. Huang et al. reported that the activity of KDM1A is essential for maintaining the stemness of cancer stem cells. Therefore, KDM1A inhibitors restore the sensitivity of sorafenib-resistant HCC cells to sorafenib in vivo by inhibiting the Wnt/β-catenin signaling pathway [222]. The KDM1A inhibitor currently comprises TCP, ORY-1001, GSK-2879552, IMG-7289, INCB059872, CC-90011, and ORY-2001197 [223]. In addition, the combination of HDAC2 inhibitor CAY10683 and sorafenib significantly slowed down the growth of drug-resistant tumors [138]. Therefore, it is evident that while these drug resistance mechanisms are all mediated by histone modifications, the distinct regulatory factors involved lead to variations in the drugs employed. This forms the epigenetic basis for personalized treatment.

Precision oncology frameworks ultimately aim to circumvent therapeutic vulnerabilities arising from tumor diversity through systematic molecular profiling of resistance mechanisms. Accelerating the empirical validation of HCC-associated epigenetic networks and computational modeling of tumor heterogeneity will enable clinically actionable precision therapeutic paradigms.

### Epigenetic therapy and nanomedicine

Nanomedicine developed based on tumor characteristics and mechanisms is ushering in a new era of cancer therapy. Nanomedicine refers to drug delivery systems designed and fabricated using nanotechnology, typically ranging in size from 10-200 nm. By encapsulating drug molecules in nanocarriers (such as liposomes, polymeric nanoparticles, micelles, dendrimers, and inorganic nanoparticles) and modifying these carriers to enable precise drug release at tumor sites, nanomedicine can significantly improve drug solubility, stability, pharmacokinetic properties, and targeting capability. This leads to enhanced therapeutic efficacy while reducing side effects [224–226]. Combination therapy derived from nanomedicine represents a promising strategy for treating drug-resistant HCC. For instance, Zhou et al. designed a miRNA-loading PLGA-PLL (polylactic acid-glycolic acetic copolymer grafted hyper-branched polylysine) carrier and synthesized a novel polymer nanoparticle (Ab-miR-NPs) by modifying it with GPC3 antibody hgc33 and loading it with miR let-7b-5p. Ab-miR-NPs increased the sensitivity of sorafenib-resistant HCC by interfering with the expression of IGF1R and inhibiting the activity of Ras/Raf and PI3K/Akt signaling pathways associated with cell resistance [227].

In summary, nanomaterials demonstrate vast application potential in the field of dual-drug delivery. By integrating smart responsiveness and targeted modification strategies, nanomedicine is expected to provide more effective and safer therapeutic solutions for drug-resistant HCC treatment.

## Summary and prospects

Overall, the current therapeutic landscape for HCC remains inadequate, with drug resistance representing a critical challenge. Extensive research has highlighted the pivotal role of epigenetic modifications in conferring resistance to both sorafenib and lenvatinib. In this comprehensive review, we have systematically reviewed these mechanisms from the abnormal regulation of ncRNA, DNA methylation, RNA methylation, and histone modification. These epigenetic modifications affect the physiological behaviors, including PCD inhibition, metabolic reprogramming, the formation and maintenance of drug-resistant cells, cell proliferation signaling pathway dysregulation, and abnormal HCC transport process, ultimately leading to resistance to sorafenib and lenvatinib. Our review also explores the therapeutic potential and research directions of epigenetic interventions from multiple perspectives, including strategies targeting epigenetic drug resistance mechanisms, the influence of resistance subtypes, and tumor heterogeneity on treatment efficacy. The proactive advancement of personalized treatment represents a critical future clinical direction, despite being a highly demanding approach requiring substantial technological and financial resources. We further discuss the emerging promise of nanomedicine in overcoming therapeutic resistance, an innovative strategy poised to benefit patients with advanced-stage malignancies. However, current research on sorafenib/lenvatinib drug resistance mechanisms remains incomplete, and epigenetic therapy represents an emerging field. Based on these limitations, we propose critical unresolved questions and outline promising research directions in this area.

(1) Mechanistic studies may involve potential conflicts and biases in experimental design or data analysis. First, the use of small clinical sample sizes in some mechanistic studies, such as the study by Yu et al., which included only 20 cases [113]. Second, the reliance on induced drug-resistant hepatocellular carcinoma cell lines as resistance models rather than clinical drug-resistant tumor models; the dominance of preclinical studies raises concerns about translational validity, as mouse models cannot fully replicate human conditions. Third, confirmation bias may occur in data interpretation, with some studies selectively reporting positive correlations between drug resistance mechanisms and resistant phenotypes. For instance, Li et al. reported that lncRNA PVT1 promotes sorafenib resistance by positively regulating GPX4 [63], which contradicts the earlier finding by He et al. that PVT1 directly interacts with miR-214-3p to inhibit GPX4 [228]. Therefore, further investigation is warranted to determine whether lncRNA PVT1 exerts a negative regulatory effect on drug resistance.

(2) Although preliminary studies have identified shared drug resistance mechanisms and therapeutic targets between lenvatinib and sorafenib, significant unexplored aspects remain in this field, warranting further mechanistic and translational investigations. Targeting these shared therapeutic resistance mechanisms or common molecular targets may enable dual sensitization of HCC to both sorafenib and lenvatinib. However, this hypothesis requires rigorous validation through further investigation. Successful clinical translation of such therapeutic strategies could provide substantial survival benefits for patients with advanced-stage HCC.

(3) Epigenetic complexity arises from multilayered dynamic regulation and tumor heterogeneity. Drug resistance mechanisms form intricate regulatory networks through interconnected interactions, exacerbating therapeutic challenges. Future research on epigenetic drug resistance should extend beyond mechanistic studies and epigenetic drug development to address challenges posed by complex regulatory networks and tumor heterogeneity. Combination therapies must evolve beyond pairing targeted agents with resistance-reversing drugs; they should also pay attention to investigate multi-epigenetic drug combinations to maximally counteract resistance mechanisms. It is noteworthy that nanomedicine represents a promising alternative strategy to overcome these barriers.

(4) Epigenetic therapies show promise but face fragmented mechanistic understanding and early-stage development, with most agents still experimental. Expanding approved drugs and validating their HCC efficacy is critical. Approved epigenetic therapies show variable efficacy due to tumor heterogeneity and resistance mechanisms. Advancing understanding of resistance pathways and patient-specific responses could address these limitations. Combination strategies pairing epigenetic drugs with targeted therapies show substantial potential to overcome resistance, though multicenter trials are needed.

In a word, the translation of existing research findings into practical and usable clinical drugs is a central focus of future research endeavors. With continued discovery of drug resistance mechanisms, we anticipate an expansion of comprehensive clinical treatment options in HCC, which could significantly benefit patients with mid-stage and advanced HCC.
